# Using mHealth to improve adherence and reduce blood pressure in individuals with hypertension and bipolar disorder (iTAB-CV): study protocol for a 2-stage randomized clinical trial

**DOI:** 10.1186/s13063-022-06449-9

**Published:** 2022-06-29

**Authors:** Jennifer B. Levin, David J. Moore, Colin Depp, Jessica L. Montoya, Farren Briggs, Mahboob Rahman, Kurt C. Stange, Douglas Einstadter, Celeste Weise, Carla Conroy, Joy Yala, Ethan Radatz, Martha Sajatovic

**Affiliations:** 1 Department of Psychiatry, Case Western Reserve University School of Medicine, 10524 Euclid Ave. 7th floor, Cleveland, OH 44106 USA; 2 Neurological and Behavioral Outcomes Center, University Hospitals Cleveland Medical Center, Cleveland, OH USA; 3 Department of Psychiatry, Case Western Reserve University School of Medicine, Cleveland, OH USA; 4 HIV Neurobehavioral Research Program (HNRP), Department of Psychiatry, University of California San Diego, San Diego, CA USA; 5 Stein Institute for Research on Aging, Department of Psychiatry, University of California San Diego, San Diego, CA USA; 6 Department of Population and Quantitative Health Sciences, Case Western Reserve University School of Medicine, Cleveland, OH USA; 7 Division of Nephrology and Hypertension, University Hospitals Cleveland Medical Center, Case Western Reserve University School of Medicine, Cleveland, OH USA; 8 Department of Family Medicine and Community Health, Case Western Reserve University School of Medicine, Cleveland, OH USA; 9 Center for Community Health Integration, Case Western Reserve University School of Medicine, Cleveland, OH USA; 10 Center for Health Care Research and Policy, MetroHealth Medical Center, Cleveland, OH USA; 11 Department of Medicine, Department of Population and Quantitative Health Sciences, Case Western Reserve University School of Medicine, Cleveland, OH USA

**Keywords:** Medication adherence, Hypertension, Bipolar disorder, Mobile intervention, Text messaging, Habit formation, Antihypertensives, Blood pressure

## Abstract

**Background:**

Cardiovascular disease in individuals with mental health conditions such as bipolar disorder is highly prevalent and often poorly managed. Individuals with bipolar disorder face significant medication adherence barriers, especially when they are prescribed multiple medications for other health conditions including hypertension. Poor adherence puts them at a disproportionate risk for poor health outcomes. As such, there is a need for effective interventions to improve hypertension medication adherence, particularly in patients that struggle with adherence due to mental health comorbidity.

**Methods:**

This 5-year project uses a 2-stage randomized controlled trial design to evaluate a brief, practical adherence intervention delivered via interactive text messaging (iTAB-CV) along with self-monitoring of medication taking, mood, and home blood pressure (*N* = 100) compared to self-monitoring alone (*N* = 100). Prior to randomization, all participants will view an educational video that emphasizes the importance of medication for the treatment of hypertension and bipolar disorder. Those randomized to the texting intervention will receive daily text messages with predetermined content to address 11 salient domains as well as targeted customized messages for 2 months. This group will then be re-randomized to receive either a high (gradual taper from daily to weekly texts) or low booster (weekly texts) phase for an additional 2 months. All participants will be monitored for 52 weeks. The primary outcomes are systolic blood pressure and adherence to antihypertensive medication as determined by a self-reported questionnaire and validated with an automated pill-monitoring device. Secondary outcomes include adherence to bipolar disorder medications, psychiatric symptoms, health status, self-efficacy for medication-taking behavior, illness beliefs, medication attitudes, and habit strength.

**Discussion:**

This study specifically targets blood pressure and mental health symptom control in people with bipolar and includes implementation elements in the study design intended to inform future scale-up. Promising pilot data and a theoretical model, which views sustained medication-taking behavior in the context of habit formation, suggests that this remotely delivered intervention may help advance care for this high-risk population and is amenable to both scale up and easy adaptation for other groups with poor medication adherence.

**Trial registration:**

The study was registered at ClinicalTrials.gov (NCT04675593) on December 19, 2020.

**Supplementary Information:**

The online version contains supplementary material available at 10.1186/s13063-022-06449-9.

## Introduction

### Background and rationale

Cardiovascular (CV) disease in individuals with mental health conditions such as bipolar disorder (BD) is highly prevalent and often poorly managed [1]. Improving adherence to medications that reduce CV risk in individuals with BD is of critical public health importance [2]. A practical, technology-facilitated and patient-centered adherence intervention has the potential to improve adherence to antihypertensive medications and reduce systolic blood pressure (SBP) in poorly adherent individuals with BD and hypertension (HTN).

BD is a chronic mental health condition that affects approximately 2% of the population and which causes substantial medical, psychiatric, and financial burden [3–6]. In 2015, total economic burden of BD in the USA was estimated at $202.1 billion, corresponding to an average of $81,559 per individual/year [7]. In spite of spending more for their care, individuals with BD have a life expectancy that is shortened by 10–30 years [1, 8–11] with CV disease being the leading cause of death [12]. Between 25 and 45% of those with BD suffer from HTN [13] and non-adherence to antihypertensives, estimated to occur in 50–80% of patients [14, 15], is a significant risk of acute CV events.

Improved blood pressure control with antihypertensive drug therapy has led to dramatic improvements in US national rates of events such as heart attack and stroke [16–19]. However, individuals with BD have multiple adherence barriers that prevent them from getting the maximum benefit from available treatments. Barriers include negative attitudes about medications, lack of information, forgetting, poor organization, ineffective routines, substance use, and low self-efficacy for medication-taking behavior [20–23]. Primary care clinicians may find HTN management of patients with BD challenging given their trouble using standard medical systems appropriately with high no-show rates for medical appointments and inappropriate reliance on crisis-based care.

The Systolic Blood Pressure Intervention Trial (SPRINT), a large and rigorous longitudinal study, contributed to a change in treatment guidelines for HTN by the American Heart Association (AHA) and American College of Cardiology [24]. SPRINT showed that a treatment strategy designed to lower systolic blood pressure to less than 120 mm Hg was associated with lowering the risk of CV events by 25% and overall mortality by 27% compared to standard care with a target SBP of 140 mmHg [25]. Reaching this lower target required an average of 3.0 antihypertensive medications compared to 1.9 medications for the standard care group [25].

Medication treatment regimen complexity, including polypharmacy, is a risk factor for non-adherence and may be even more salient for individuals with BD who are likely to be on multiple medications [21, 26–28]. Polypharmacy is associated with worse adherence in treatment-resistant hypertensive patients [29]. In a large retrospective study, 42% of a hypertensive cohort that started out medication adherent became poorly adherent (defined as taking less than 80% of prescribed medications) during a 52-week follow-up period. In this same study, reduced adherence to diuretics was the most common pattern identified [29]. Studies suggest that those who need the most aggressive antihypertensive treatment become increasingly less adherent over time [30].

There is a need for practical and effective interventions to improve HTN medication adherence, particularly in patients that face challenges to their medication adherence due to mental health comorbidity. Two recent reviews of adherence interventions indicate that there is no single intervention that has strong evidence for improving antihypertensive drug adherence [31, 32]. For many patients, poor adherence is a matter of “can’t” rather than “won’t” due to forgetting to take medications or having lifestyles that make it hard to incorporate medication-taking into daily routines. Prospective memory, or the ability to remember to engage in a behavior in the future, tends to be impaired in those with BD due to executive functioning deficits such as planning and cognitive flexibility [33–35]. Thus, even when an individual has the intention to take medication, they may lack the planning or organizational abilities to do so consistently [34]. The literature indicates that forgetfulness and lack of routines is the most important reason for non-adherence [36]. Furthermore, for individuals with BD, medication regimens may be relatively complex, involving treatments for HTN as well as mood stabilizing or antidepressant drugs. Yet, there are few effective interventions to enhance adherence and improve outcomes in BD [21, 32] and those that do exist do not simultaneously target non-psychotropic non-adherence.

In summary, despite strong evidence for antihypertensive medications in reducing CV risk on a population level, adherence remains poor for many individuals, especially in people with mental illnesses such as BD. An effective adherence enhancement approach for patients with BD needs to (1) take into account the need for multiple medications, (2) address the additional adherence challenges that exist such as difficulty in planning ahead and in establishing stable healthcare routines, and (3) simultaneously target non-psychotropic and psychotropic medication adherence. Approaches that are brief, practical, and which can be embedded into standard medical care settings are of substantial public health significance as they can improve outcomes for high-risk patients and may serve as a template to improving antihypertensive adherence in other challenging patient sub-groups.

### Trial design

This 5-year project uses a 2-stage parallel group, two-arm, superiority randomized controlled trial (RCT) design with a 1:1 allocation ratio to evaluate a brief, practical adherence intervention called Individualized Texting for Adherence Building – Cardiovascular (iTAB-CV), delivered via interactive text messaging, along with self-monitoring (SM) compared to SM alone. The underlying premise is that improving adherence to antihypertensives in BD is likely to be an efficient path to improving health outcomes in a vulnerable population. The intervention is suitable for primary care or mental health settings and has potential for broad scale-up. Findings on the relationship between habit strength, medication attitudes, mood, and adherence in this project will be generalizable to other populations.

In considering the appropriate comparator to iTAB-CV in this efficacy trial, based on Mohr et al.’s seminal paper on the selection of a control condition for RCTs [37], SM will be composed of monitoring pills, blood pressure (BP), and mood as it is generalizable to standard clinical care. Each of these elements has been shown to have some efficacy in lowering SBP [38] and would allow us to determine whether iTAB-CV is efficacious above and beyond improvement yielded from implemented current clinical recommendations. The SM process for all participants involves instructing them to monitor their medication taking using an automated pill-monitoring device, take their BP weekly with a home BP monitor, and rate their mood weekly in response to a text reminder.

## Objectives

This study has four specific aims: (1) to test the efficacy of iTAB-CV + SM compared to SM alone to improve medication adherence using a RCT design, (2) to test the efficacy of iTAB-CV + SM compared to SM alone to lower SBP, (3) to explore outcomes as a function of high vs. low booster intensity compared to SM alone at 6 months (2 months post-intervention) to inform future tailoring, and (4) to explore factors that impact adherence decay between iTAB-CV + SM vs SM alone at 9 and 12 months.

Exploratory analyses will capture whether frequency of texts, depression, engagement, medication attitudes, and self-efficacy serve as moderators of longer-term outcome (6 months). Findings from this data will inform future patient-centered refinements to the intervention delivery format that will match intervention content and frequency to patient needs. Analyses will investigate the trajectory of outcomes once iTAB-CV is discontinued and whether those randomized to iTAB-CV + SM maintain better medication adherence and lower SBP compared to SM alone at 9 and 12 months.

Additional exploratory mediation analysis of adherence will include habit strength as a mediator and identification of variables that enhance (i.e., greater care engagement) or impede (i.e., depression or other psychiatric symptoms) adherence. Exploratory analysis will help characterize non-responders and determine needed adaptive modifications in frequency and length of treatment. To enhance future implementation efforts, the project will be informed by input from an advisory board made up of key stakeholders including patients, family members, providers, and administrative staff who can help inform how the intervention might fit into existing clinical workflows.

## Methods

The study methods follow the SPIRIT reporting guidelines [39] and are verified with a completed SPIRIT checklist.

### Participants

Because many individuals with BD tend to rely on public-sector care, recruitment will target diverse locations including the community, an academic medical center, local primary care settings that provide care to individuals of limited resources, and community mental health clinics. The research team will network with key representatives, such as staff from federally qualified health centers in the region, community centers, and local community mental health clinics to obtain appropriate referrals and recruit through advertising as approved by the Institutional Review Board (IRB). Electronic health records at University Hospitals will also be queried to determine a list of potential participants, who will be contacted by opt-out letters and follow-up phone calls. Pilot data suggest that a substantial proportion of participants will be minorities. Research coordinators will consent and enroll up to 200 RCT participants at an academic medical center in an urban setting and up to 12 stakeholder advisory board (SAB) members for a total of 212 participants. It is expected that most of the patient SAB members will be referred from clinicians both at the academic medical center in an urban setting as well as other local organizations serving persons with these health conditions.

Participants will have a self- or clinician-reported diagnosis of HTN, have a clinical diagnosis of BD determined at screening, be at least 21 years of age, be less than 80% adherent with their HTN medicine, and be able to provide written informed consent and participate in study procedures. Along with non-adherence, participants must have an average elevated SBP defined as at least 130 mmHg at the time of screening. Participants must also have cellular phone with texting capabilities in order to receive the intervention. Individuals at high immediate risk for suicide, those who are monolingual and non-English speaking, and individuals with an upper arm circumference greater than 50 cm will be excluded. The rationale for the latter exclusion stems from the absence of validated home blood pressure monitors for individuals with an upper arm circumference greater than 50 cm. All study participants will continue to receive services with their regular medical clinicians and providers at all sites.

**Fig. 1:**
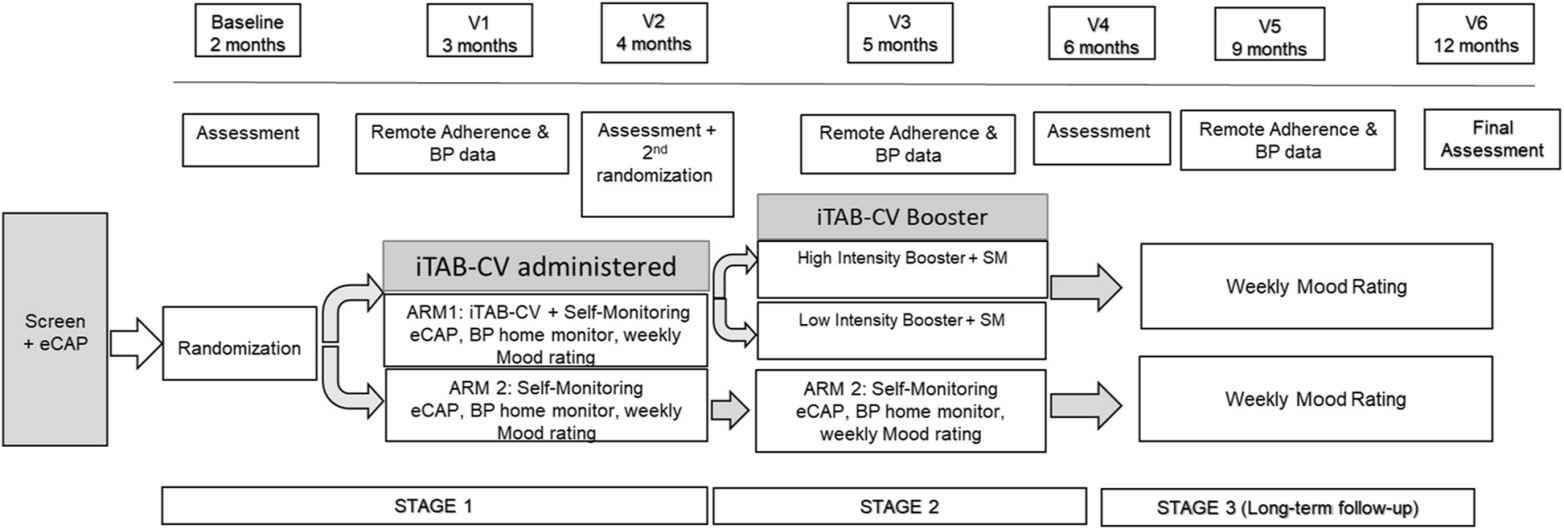
Two-stage RCT design

As seen in Fig. 1, the entire observation duration will be 12 months. In stage 1, after consenting and passing screen, participants will receive and be trained on the use of an automated pill-monitoring device called eCAP™ (manufactured by Information Mediary Corporation, Ottawa, ON, Canada) to track use of their antihypertensive medication. Participants will use the eCAP™ for 2 months until coming in for their baseline assessment. At that time, they will be randomized on a 1:1 basis to participate in either iTAB-CV + SM or SM alone. Both interventions will be provided for 2 months with an interim phone assessment for adherence. At the end of this 2 month stage, participants will come in for an in-person assessment.

In stage 2, following the month 4 assessment, those in iTAB-CV + SM will be re-randomized to receive either a high intensity (starts off with 1 reminder per day and tapers down to 1 reminder per week over the course of 2 months) or low intensity (1 reminder per week) booster. Adherence will be assessed by phone at month 5 as an interim measurement and an in-person assessment will occur at month 6. In stage 3, participants will be followed for another 6 months without intervention with one interim phone assessment and a final in-person assessment at month 12.

As this is a group of participants who may be particularly difficult to engage and retain in longitudinal research studies, there will be a number of measures taken to optimize retention. These include scheduling visits at maximally convenient times for participants, conducting visits remotely when possible, allowing for frequent breaks as needed during the procedures, hiring and training staff that will be flexible and attentive to the individual’s needs, and frequent opportunities for questions and feedback. Participants will also receive small items with the study logo of nominal monetary value but that will remind them of study participation such as a stress ball in the shape of a heart, pen to log adherence, and hand-sanitizer. Reminder calls will be made prior to assessments. Should there be challenges in meeting enrollment goals, the study team will request input from the SAB in additional procedures and processes that might optimize enrollment. Participants in all treatment groups will be compensated for assessments.

### Study setting

Research procedures will be performed either in the principal investigators’ (PIs) research offices, which are located at an urban academic medical center in Cleveland, Ohio, USA, or, given safety concerns in the COVID-19 era, can be conducted remotely. A list of study sites can be obtained by contacting the corresponding author. The consent process may be conducted using a secure videoconferencing system (Zoom) and via a secure data entry and storage system called Research Electronic Data Capture (REDCap). Other study procedures and visits may also be conducted via videoconferencing, phone, and/or via REDCap survey (individual link emailed directly to the participant) in the event an in-person visit is not possible. In addition, SAB meetings will be conducted remotely.

### Stakeholder advisory board (SAB)

To ensure that iTAB-CV meets the needs of patients and clinicians, and fits into workflows and operations of clinical systems, the investigators will obtain input from a SAB made up of patients, family members, clinical providers, and health system administration representatives. The SAB will meet for an hour approximately 3 times for the first 6 months of the project and then once yearly for a total of 7 meetings over the course of 5 years to (1) identify modest refinements of the intervention that will address barriers and facilitators to being able to implement the project and intervention in the RCT, (2) support recruitment/retention efforts, and (3) gather process data during the RCT regarding how future implementation and dissemination efforts might enhance reach and adoption of iTAB-CV into clinical settings. Information obtained from these local stakeholders will also help the investigators to develop a robust recruitment strategy and a set of practices that will maximize engagement and retention of participants.

The study team will collect demographic information from SAB members at the time of consent. Study staff will also collect information on SAB members’ lived or professional experience with BD/HTN and feedback on the program and implementation. SAB meetings will be audio/video recorded. Finally, after each meeting, study staff will survey SAB members on their satisfaction with the SAB meeting.

### Equipment and devices

All participants will receive a home BP monitor at screening and undergo training on its use by study staff based on a protocol provided by the British Hypertension Society (BHS) and the National Institute for Health Research [40]. They will also receive written information including a description and pictorial presentation of how to take and record BP readings. The monitors used in this study are the Omron® 3 Series BP7100 monitors and the Omron® 7 Series Wrist BP6350 monitors for those whose arms cannot fit a standard arm cuff (manufactured by Omron Corporation, Shimogyo-ku, Kyoto, Japan). Both models are validated and approved for home use [41], and both have a memory function to record and display several recent BP readings.

All participants will have remotely administered pill monitoring via the eCAP™ which records pill bottle openings to a secure, cloud-based database. Pill bottles equipped with eCAPs™ are capable of storing a 90-day supply of one medication. Once the eCAP™ is activated by the Certiscan® Secure Reader, the eCAPs™ record each time they are opened. The data are uploaded to the cloud remotely by scanning the eCAP™ via an app on the participant’s personal smartphone or by using the Certiscan® Secure Reader. The eCAP™ will be distributed to participants when they meet all inclusion criteria for enrollment. The measurement of adherence following the introduction of electronic pill monitoring has been shown to influence medication adherence (Hawthorne effect) initially [42]. In the pilot study, adherence improved in the 4 week period between screen and baseline. Therefore, the pre-intervention phase will be extended to 8 weeks during which both treatment arms will just be monitored via eCAP™. It is anticipated that 4 weeks is long enough for return to regular medication taking behavior, so the past month and past week adherence will be gathered from the second month. Participants are incentivized to return the eCAP™ at the end of their study participation.

### Educational video

Following baseline assessments and prior to randomization, all participants will watch a brief educational video about the symptoms, risks, and the important role of medication in the treatment of both HTN and BD. The content parallels that which was presented during the pilot delivered by the PI using a Powerpoint. The content was derived from publically available information disseminated by the American Heart Association on HTN [24] and by the National Institute of Mental Health on BD [43] and reviewed for accuracy and understandability by experts in internal medicine and psychiatry from the research team.

### Intervention

Individualized Texting for Adherence Building – Cardiovascular (iTAB-CV) for individuals with BD and HTN is an adherence intervention utilizing cognitive and behavioral principles based on a modified version of the Attitude-Social Influence-Efficacy theoretical model [44]. The iTAB-CV content aims to increase positive medication attitudes, draw on social support and comparison, and strengthen self-efficacy for medication taking behavior. Participants in the intervention group will receive texts that include educational/motivational content and be asked to respond whether the medication was taken. While the pilot presented iTAB-CV in 2 stages, the first with educational/motivational content but no reminders and the second with both educational content and reminders, participants indicated that they viewed the educational texts as reminders. As such, in the current trial, there will be no explicit medication reminders. The timing of the messages will correspond with their first antihypertensive medication dose of the day as determined by the participant and sent within a 1-h window (half hour before or after) of the scheduled time. The reason for the variability is to keep the system new and reduce the likelihood of ignoring messages that arrive at exactly the same time every day.

iTAB-CV texts will be administered for a total of 4 months. Participants in the intervention group will receive the texts once a day for 2 months following baseline, then following a second randomization of the iTAB-CV group, will receive the texts either once weekly or gradually tapered down from daily to once weekly for another 2 months.

In iTAB-CV, sending texts is automated via a central server, which provides an advantage over more labor intensive and costly approaches. The content of the iTAB-CV texting system messages combines predetermined content in 11 domains with customized message content. The order of the messages is prescheduled such that all participants will receive the same content on the same day of the study with placeholders for customized content. The customization ensures that texts are personal, salient, and do not become stale. The 11 domains were derived from the original focus groups conducted in phase I of the R21 and in consultation with the study co-authors with experience using the iTAB-CV system and clinical specialists [45]. The specific text stems were categorized into existing domains by 3 independent research staff (see Table 1).

**Table 1:** Domains and sample stems

Domains	Sample stems
Hypertension knowledge	Blood pressure medication is a NECESSARY and EFFECTIVE way to lower blood pressure.
Bipolar disorder knowledge	BD^a^ results from a problem in the brain’s ability to regulate chemicals or ‘neurotransmitters’.
Benefits of blood pressure medication	Blood pressure meds help lower blood pressure and improves heart health.
Benefits of BD medication	BD meds help me function better and make it less likely that I will get depressed.
Making peace with medication	Although I may wish I did not need meds, accepting that I do makes me stronger!
Social support	My physical and mental health impacts others; I should take my meds for them.
Self-efficacy	I believe that if I take my meds daily, my health will improve.
Medication routines	Keep meds somewhere I will see them.
Spiritual	God grant me the serenity to take care of my health by taking my meds.
Self-esteem	I am special. I deserve to be healthy!
Social comparison	I would encourage others with BD to take medication to stabilize their mood.
Custom	Lisa wants me to be healthy so we can build a brighter future together!

^a^*BD* bipolar disorder

iTAB-CV is personalized with texts that are specific to the person, medication, and timing. Motivational interviewing demonstrates that change is more effective when patients use their own words to commit [45] and personalization of messages has been effective in changing behaviors in other diseases such as HIV-infected individuals with BD and in diabetes [46–48]. Moreover, iTAB-CV uses the participant’s own words for both reminders and reinforcers. Personalizing reminders and reinforcers offer an advantage over previous studies that have focused on medication timing and dosing but did not tailor the reminder itself [46–49]. Participants will be encouraged to write their own adherence reminders (e.g., I want to stay healthy so I can care for Sam and Jane for many years!”) as well as reinforcement stems within the character limits (e.g., “Susie is so proud of you for taking your meds!”). Participants will choose their preferred name for HTN and BD to be used in some of the text stems and will have the opportunity to opt out of 2 of 12 categories: Social Support and Spirituality. In those cases, Social Comparison texts, customized texts, and alternate texts will be substituted.

**Fig. 2:**
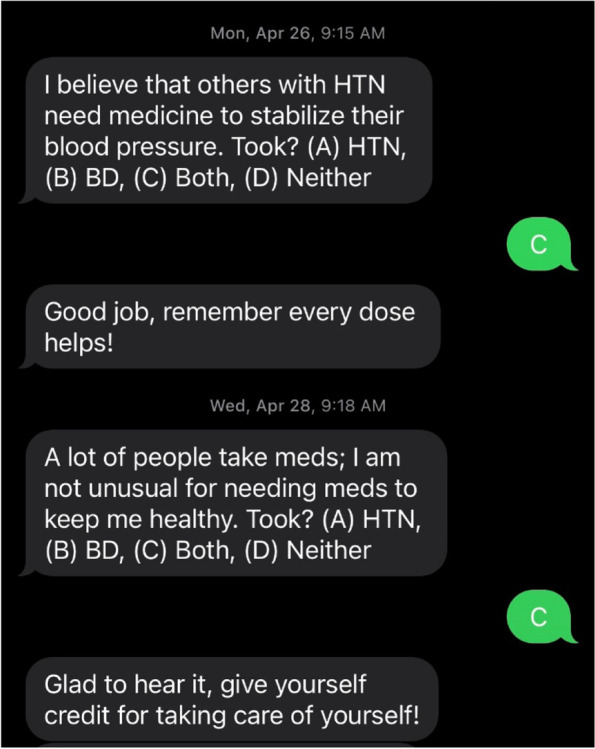
Sample text messages

Adherence texts will be followed by a question asking whether medication was taken. All responses will be followed by immediate reinforcement either to reinforce medication taking behavior or to reinforce engagement with the system if response indicates medicine was not taken (see Fig. 2 for sample text and reinforcement exchange).

**Fig. 3:**
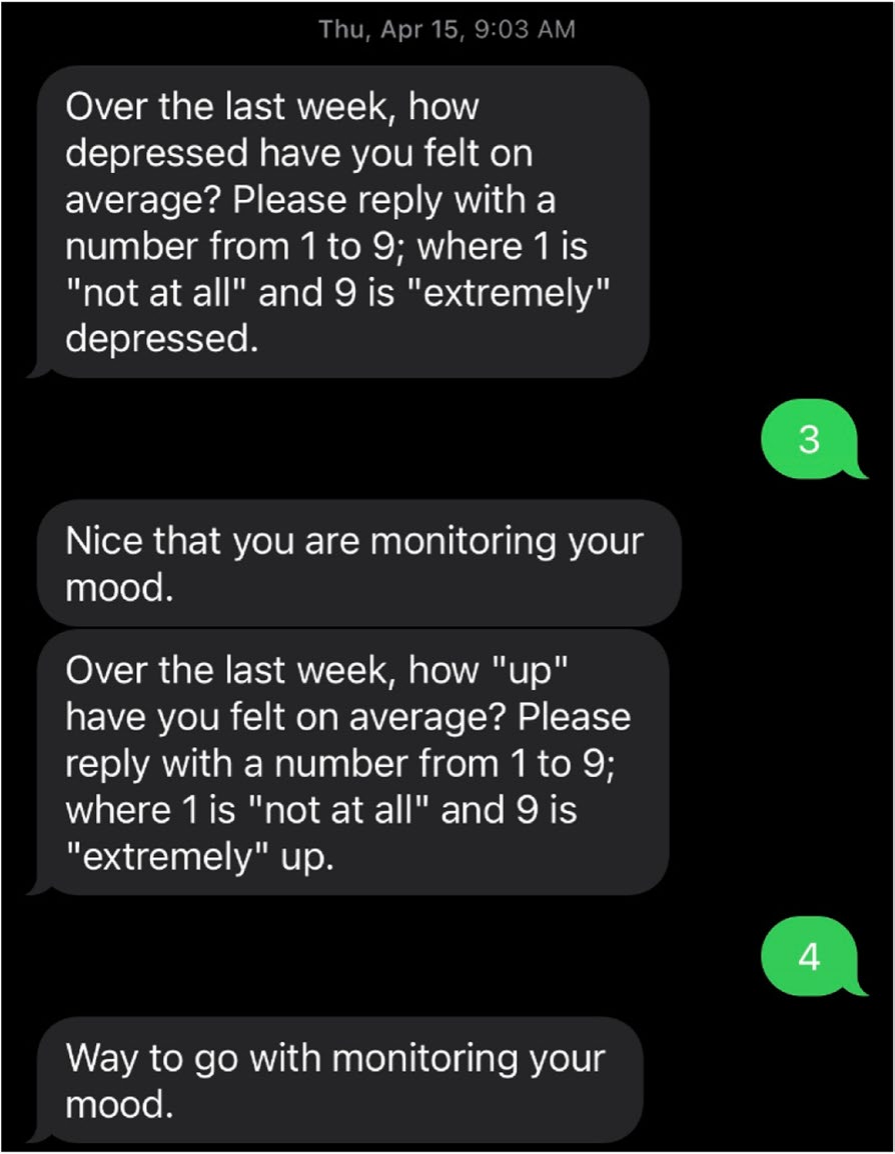
Sample mood rating messages

Participants in both treatment arms will receive mood rating messages from the iTAB-CV system once a week from baseline to the end of the study. They will be asked to rate how depressed they have felt on average (where 1 is “not at all” and 9 is “extremely” depressed) as well as how “up” or “irritable” they have felt on average (where 1 is “not at all” and 9 is “extremely” up or irritable) (see Fig. 3 for sample mood rating exchange). This method for collecting mood ratings was found to be feasible and acceptable in a study evaluating the use of text messaging for measuring change in depression over the course of a RCT [50]. Unlike in the pilot study where participants completed daily mood ratings, in this trial, we decided that more information regarding mood, including mixed symptoms, would be gained from two separate Likert scales and that weekly ratings would increase the message freshness and thus responsiveness. The automated system will also send all participants medication refill reminders for their antihypertensive medication once a month from baseline to the end of the study.

Responses to texts and lack thereof will be recorded within the iTAB-CV system. Multiple responses to the same message will yield an automated text indicating that the message has already been received, and inaccurate responses (i.e., not in the requested format) will return an automatic message saying that a given text was not understood by the system. Similarly, there is an automated texting sequence to address lack of responsiveness. After 3 consecutive days of missed messages, the automated system will send an alert to a mobile interventionist (MI) for them to carry out follow-up phone calls to support troubleshooting. There is also a texting help email provided so the participant can correct any errors or solicit help from an MI.

### Mobile interventionists

Two MIs with at least a bachelor’s degree and experience working with individuals with serious mental illness and comorbid chronic medical conditions will be assigned to meet with participants based on MI and participant availability. The MIs will be trained and supervised by one of the PIs. The MIs will meet with participants between 30 and 60 min to personalize and train them on the use of the automated texting system. For individuals randomized to the intervention iTAB-CV + SM, MIs will use a structured interview, developed in the pilot study, targeting the 11 domains to identify what motivates them to take medications for HTN and BD, to personalize their adherence texts and reinforcers, and to determine whether they would like to opt in or out of the Spirituality and Social Support domains. The MI will also set up participants randomly assigned to SM in the system to receive their mood rating and refill reminder text messages. All participants will be trained on responding to the text messaging system by their assigned MI. To decrease potential bias, these interventionists will not be involved in the assessment component.

Participant and MI interview and iTAB-CV system setup for all participants will be video/audio recorded and a minimum of 25% of them will be viewed and assessed for fidelity by one of the co-PIs. The fidelity monitor will use a fidelity scale adapted from previous work using the iTAB-CV platform, discussing discrepancies and retraining mobile interventionists as needed. The fidelity monitor will also review at least 25% of the records to evaluate consistency between the structured interview set up form and the iTAB-CV system itself. The fidelity scale will be completed in its own REDCap project.

### Harm risk reduction

If at any point a participant presents with what are deemed to be unsafe BP readings (i.e., systolic BP greater than or equal to 180 mmHg or diastolic less than 90 mmHg), the study PIs or internist-investigator will be notified and the participant will be advised to seek immediate medical care through their primary care provider, urgent/express care, or emergency department. There is an automated texting sequence in the iTAB-CV system to address responses to mood texts indicating severely low or high mood and provide the participant with information on how to access immediate emergency services. An automated text sent to the participant following report of extreme moods (7–9 on either depression or elevated/irritable mood) to engage their supports and follow their safety plan. If there are 2 such consecutive severe mood ratings, an automatic email will be sent to the research assistant (RA) who will follow-up by phone with the participant. If during the course of the assessment, a participant communicates to the rater or investigator that they may be in immediate danger or at acute risk of harm to self or others (for example, reporting a suicide plan), the study staff interacting with the individual will immediately notify (1) the patient’s clinician and (2) one of the mental health clinician co-PIs so that all available and appropriate measures may be taken to ensure the prompt safety and most appropriate care setting for the patient, including possible termination of study participation.

### Randomization

The study biostatistician will generate the allocation sequence and randomization lists will be computer-generated and integrated into a REDCap randomization project. Members of the study staff will not have access to the sequence prior to assignment. Stratified randomization will be employed to ensure that equal numbers of iTAB-CV + SM and SM patients are balanced with respect to sex and BD severity as reflected by BD type (type 1 vs type 2). The second randomization (high or low booster) will also be balanced with respect to sex and BD type. The MIs will randomize participants to an intervention at baseline using the REDCap project with pre-populated randomization criteria. The MIs will also conduct the second randomization of the iTAB-CV + SM group to booster, which is set up in the iTAB-CV texting system. RAs who complete assessments will be blind to condition by not having access to the randomization module or iTAB-CV system and administering assessments relevant to both arms regardless of assignment. Only the MIs are aware of randomization assignment and they do not carry out any assessments. While all efforts will be made to keep RAs blind to treatment condition, participants are not blinded to condition and thus could unintentionally reveal information to the RAs during an assessment or outreach by an RA in response to extreme moods. Given that this is not a drug trial and risk is low for both groups, there are no plans for intentional unblinding.

### Assessments and outcomes

Assessments for both iTAB-CV + SM and SM alone will include evaluation of medication treatment adherence, psychiatric symptoms, health status, self-efficacy for medication taking behavior, illness beliefs, medication attitudes, and habit strength for medication taking behavior. One-on-one assessments will be conducted at 5 time points over a 12-month time period. Adherence and BP will be measured at each of the 5 one-on-one assessments. There will be 3 additional remote/phone assessments which will collect adherence and BP data only. Individuals who drop out of the intervention, and who agree, will be followed up with outcomes assessments over the same 12-month time period that they would have been evaluated had they remained in the study.

Table 2 shows the variable constructs, how they will be measured, mode of data collection, and time points, following the structure of a SPIRIT figure. Demographic and clinical variables measured at baseline will include age, sex, race/ethnicity, education, marital status, and health status. Assessment visits include one-on-one screening, baseline (2 months), V1 (3 months; by phone), V2 (4 months), V3 (5 months; by phone), V4 (6 months), V5 (9 months; by phone), and V6 (12 months). All assessments will be done by a RA trained by the PIs with pre-established and documented reliability standards. Training will use video clips of non-study volunteers or actors. It is expected that patient assessment will require approximately 90 min for the screening assessment and approximately 60 min for the follow-up assessments. Study visits designated as one-on-one may take place in person, via phone or via video conferencing as needed.

**Table 2:** Study instruments and assessments schedule

Construct	Measurement	Mode	Screen	BL	V1 phone	V2	V3 phone	V4	V5 phone	V6
**Primary outcomes**										
Blood pressure	Systolic blood pressure	REDCap	X	X	X	X	X	X	X	X
Antihypertensive adherence	TRQ	REDCap	X	X	X	X	X	X	X	X
	eCAP™			X	X	X	X	X	X	X
**Secondary outcomes**										
Mood stabilizer adherence	TRQ	REDCap	X	X	X	X	X	X	X	X
Psychiatric symptoms	BPRS	REDCap		X		X		X		X
	MADRS	REDCap	X	X		X		X		X
Self-efficacy for medication taking	MASES-R	REDCap		X		X		X		X
Medication attitudes	BMQ	REDCap		X		X		X		X
	AMSQ	REDCap		X		X		X		X
Habit strength	SRHI	REDCap		X		X		X		X

*BL* baseline

V1 = 3 months

V2 = 4 months

V3 = 5 months

V4 = 6 months

V5 = 9 months

V6 = 12 months

The primary outcomes are SBP and adherence with antihypertensive medication. While SBP is the primary outcome for this analysis, both SBP and diastolic BP will be measured. A recently published joint Policy Statement from the AHA and American Medical Association recommend home-based BP monitoring for the most accurate assessment to minimize biased/artifact findings and yield the most valid results [51]. The joint statement states, “BP may differ considerably when measured in the office and when measured outside of the office setting, and higher out-of-office BP is associated with increased CV risk independent of office BP. Self-measured BP monitoring, the measurement of BP by an individual outside of the office at home, is a validated approach for out-of-office BP measurement. Several national and international HTN guidelines endorse self-measured BP monitoring.” In addition to optimizing accuracy, use of home-based BP evaluation will minimize potential COVID-19 exposure for study participants, most of whom are adults with multiple chronic health conditions.

At screening, participants will be trained on the use of the BP monitor following the BHS protocol [40]. Participants will be asked to take their own BP 2 times consecutively with 1 minute in between each reading and after sitting in a chair quietly for 5 min. BP readings will be collected twice a day, in the morning and evening, for 3 to 7 days while a member of the study staff observes the participant over a video platform, in person, or over the phone. Alternately, the participant can send a screen shot of the BP readings via text to study staff. Once they have obtained 12 readings total (6 in the AM and 6 in the PM), SBP will be averaged across all readings. Participants will also use these procedures to ascertain BP readings at follow-up time points. The interim BP readings will be conducted in real-time using the remote platform or by phone in real time, or if needed, will be measured beforehand and reported during the visit.

Antihypertensive medication adherence will be evaluated with the self-reported Tablets Routine Questionnaire (TRQ) [52, 53] for the past week and the past month. Self-reported adherence will be validated with the eCAP™. While the literature on measurement of adherence including the co-PIs’ own work in this area acknowledges limitations for all methods of adherence assessment, both self-report and automated pill-caps appear valid and practical for use in BD studies [21, 54].

At screening, participants will be asked about their demographic information and current medication taking behaviors to determine if they meet inclusion criteria. Participants will also undergo a diagnostic interview based on the DSM and ICD psychiatric disorders called the Mini-International Neuropsychiatric Interview (MINI) [55] to confirm BD diagnosis and assess for suicidality. Data on comorbid medical conditions will be collected based on the self-report version of the Charlson Comorbidity Index (CCI) [56]. While the original CCI includes dementia, it was excluded from the self-report version given that those with dementia would not be able to provide informed consent. Finally, participants will be administered the Rapid Estimate of Adult Literacy in Medicine-Revised (REALM-R) [57] to assess health literacy.

### Secondary outcome measures

Bipolar medication adherence will also be collected using the TRQ for medication targeting mood symptoms including lithium, anticonvulsants, antipsychotics, and antidepressants [58]. Psychiatric symptoms will be assessed using the Brief Psychiatric Rating Scale (BPRS) [59], a clinician rated measure that evaluates the spectrum of symptoms seen in individuals with BD including mania, psychosis, depression, and disorganization. The Montgomery-Asberg Depression Rating Scales (MADRS) [60], a well-validated clinician rated measure, will also be used to measure symptoms of depression and suicidality.

Researchers will utilize self-report questionnaires comprised of the Medication Adherence Self-Efficacy Scale (MASES-R) [61], the Self-Report Habit Index (SRHI) [62], the Beliefs about Medicines Questionnaire (BMQ) [63], and the Attitudes toward Mood Stabilizers Questionnaire (AMSQ) [64] to capture possible moderators and mediators to medication adherence. Dose of intervention (frequency of texts) and engagement (% texts answered) will be collected from the iTAB-CV system. Intervention acceptability/satisfaction will be assessed via questionnaire as well as open-ended process measures regarding ease of use of the system and how it can be improved. Healthcare utilization will also be measured via self-report questionnaire.

## Data collection and management

Study data will be collected and managed using REDCap, a secure, web-based application designed to support data capture for research studies providing (1) an intuitive interface for validated data entry, (2) audit trails for tracking data manipulation and export procedures, (3) automated export procedures for seamless data downloads to common statistical packages, and (4) procedures for importing data from external sources [65]. Only study team members will be able to access the REDCap project which saves to HIPAA protected servers. Data files, including analysis files, will be password protected to permit access and modification only by authorized persons. Participant names or similar potential identifiers (e.g., addresses, hospital record numbers) will not appear in any computerized database.

Lists of potential participants will be saved within the secure server and will only be accessible by study staff who have been research credentialed. For those participants who cannot be contacted, refuse participation, or otherwise do not qualify for the study, only aggregate numbers will be retained to keep track of recruitment efforts. Careful attention will be given to confidentiality, which will be maintained using subject identification (ID) codes for enrolled participants. The list that links study ID codes with subject names and all forms bearing subject names and contact information will be stored in password protected files on the institution’s secure server. Research files are not and will not be available to any unauthorized person.

Rigorous development of data collection forms, training of staff on the proper completion, and checking of data collection forms will reduce errors at the point of collection. Additional data management practices before and after entry into the database will identify potential problems or outlying values and will catch other errors on data collection forms. Data management staff will be responsible for tracking forms entered and for performing routine auditing data checks. Analytic data sets will be prepared using STATA v13.1 [66].

### Data analysis plan

For the primary intent-to-treat analyses (aims 1 and 2), linear mixed effects longitudinal analysis of adherence (TRQ past month is primary) and SBP to 4 months (V2) will be conducted. A treatment variable will be included to indicate randomization to either iTAB-CV + SM or SM alone. Secondarily, we will also consider models with longer-term outcomes. Within the longitudinal models, significant interaction of the treatment variable with time indicates that the treatments have a different course of response. The researchers will first fit models with time as a covariate and, alternatively, consider time period as a categorical variable. Time by treatment interaction will be of primary inferential interest. To account for possible imbalances across treatment groups and other sources of variation, explanatory variables such as gender, ethnicity, and BD diagnosis type (I vs. II) will be considered for inclusion in the mixed models.

Researchers will consider representing TRQ scores as binary outcomes, indicating whether or not an adherence threshold has been met (e.g., 80% adherent). Researchers will thus also consider generalized linear mixed models for binary outcomes (SAS PROC GLIMMIX), as well as fitting a longitudinal model on the difference values with baseline value adjustment. Graphical methods will be used extensively to examine distributions of residuals, identify potentially influential points, and guide data transformations to better approximate normality if warranted. Sensitivity analysis of results will be conducted by modeling a range of plausible correlation structures.

In secondary analyses for both aims 1 and 2, follow-up at 6 and 12 months (V4 to V6) will also be compared in the respective longitudinal analyses for adherence and SBP. The researchers will conduct contrast analyses of treatment by (discrete) time interaction at time periods V4 and V6 and differences from baseline in treatment dose-level subgroups (higher versus lower booster levels) at V4 and V6 using two sample *t*-tests or nonparametric Mann-Whitney tests. Additionally, the researchers will compare corresponding TRQ and eCAP™ adherence levels. Correlation between the measures will be estimated and Bland-Altman plots will be generated [67].

The researchers will model secondary outcomes over time in a similar manner as in aims 1–2 through longitudinal mixed models when appropriate. The researchers will consider generalized linear models when distributions of outcomes are not approximately normally distributed or transformations of outcome variables to normality. The researchers will also conduct exploratory moderator analyses of the explanatory variables in the primary mixed models for adherence and SBP. Moderators to be explored include depression, psychiatric symptoms, age, sex, engagement, medication attitudes, self-efficacy, and BD type I vs. II for each of the primary outcome models. For moderator analyses, in an exploratory manner, the researchers will consider the addition of interaction effects with treatment and time for each potential moderator.

For aim 3, habit strength scores will be analyzed as an outcome in a similar manner as in aim 1. The researchers will assess dose (frequency of texts), depression, engagement, medication attitudes, and self-efficacy as moderators of habit strength. The researchers will also consider habit strength as a mediator for improved longer-term adherence at 4, 6, and 12 months. The researchers will conduct mediation analyses using approaches described by MacKinnon [68] and Preacher and Hayes [69]. These analyses will involve the treatment variable and change in adherence values. Bootstrapping methods will be used to assess indirect effects, as in Preacher and Hayes [69]. The research team will also consider, as needed, use of generalized linear models through a mediation formula approach [70, 71] to estimate the direct effect of the treatment and indirect effects through the proposed mediator.

### Sample size calculations

Power analyses are based on computations from the Repeated Measures and Sample Size (RMASS) [72] with inputs estimated from prior study data. Our projected sample size is *n* = 200, with 100 participants per arm. In this power analysis, the research team will assume type I error levels of 0.025 for each of the two primary analyses, using Bonferroni correction to account for multiple comparisons. Conservatively, for this study, the researchers will assume 25% attrition based on a previous 12 month trial conducted by members of the study team with individuals with comorbid serious mental illness and diabetes [73]. For sample size requirement for aim 1, the investigators used past-month TRQ as an approximation of expected and longer-term maintenance adherence status and considered primary outcomes to be at 3 months. The investigators expect similar or even greater effect sizes at 4 months given the longer treatment duration. In the pilot study, there was 23.10% improvement in TRQ observed for iTAB-CV. The control comparator for the efficacy trial will be SM of adherence, BP, and mood. Based on the literature, no improvement in self-reported adherence was reported in a similar control arm [74]. The study staff thus assume no change in adherence. In a BD adherence RCT, 7% improvement was observed in the control arm. Hence, the investigators conservatively project a 7% mean improvement in adherence among controls. Thus, (1) there is a mean difference of 16.1% in adherence after 4 months between arms and (2) suppose autoregressive (AR(1)) model covariance structure with correlation coefficient of 0.44 and an error variance of 308, as estimated value from the data. With assumed attrition of 25%, for a 2-sided test of the treatment by time interaction effect being equal to zero, and with alpha = 0.025 and power = 0.80, the required total sample size is approximately 78.

From the pilot data, mean screen SBP was 144.81 (SD 15.46). Change from screen to 3 months with the iTAB-CV intervention saw a mean reduction in SBP of 8.78. Reductions in BP of at least 2 mmHg can significantly reduce the incidence of cardiovascular disease and its complications in both hypertensive and normotensive individuals, and reductions of the magnitude seen in this team’s pilot work is well above thresholds that are considered clinically meaningful [75]. Based on our preliminary mixed model, the researchers also observed compound symmetry correlation parameter value of 0.36. This covariance model had similar model fit as with an AR(1) model in terms of AIC/BIC criteria and parameter estimate values. The researchers will assume no SBP pressure change is observed in the SM arm and estimated error standard deviation of 15.46, as observed [38]. Finally, in a linear mixed model with participant-level random effects, with two-sided type I error of 0.025, power = 0.80 for the projected sample size of 182 participants. The study staff will target a sample of 200 for the study to allow for some deviation from assumptions. Thus, the researchers should have sufficient statistical power for the first 2 primary aims.

### Missing data

Preliminary analyses will determine factors that are associated with attrition effects. Attrition rates in the pilot iTAB-CV adherence study was 0%. In the team’s previous BD adherence studies, attrition rates were 20%. Data that remain missing despite retention efforts will be accommodated in our analyses and their impact evaluated through sensitivity analyses. The research team will study reasons for dropout and identify covariates associated with dropout by 4 months through binary regression, including the treatment variable. Differential attrition by key covariates such as age, sex, and BD diagnosis will also be examined such as through log rank tests. These models can be estimated without bias under the missing at random (MAR) assumption [76] and provide valid analysis when the covariates associated with missingness (if any) are included in the mixed model. Researchers will conduct assessment of the MAR assumption by pattern mixture models that relax the MAR assumption, while analyzing the sensitivity of treatment by time interaction effects. Researchers will also consider selection models to assess sensitivity of findings to the MAR assumption in a Bayesian Markov Chain Monte Carlo framework [77, 78].

### Data and safety monitoring board (DSMB)

As participants in the study are a vulnerable population, we recognize the need for a careful data and safety monitoring plan to ensure the well-being of the participants in this study and the scientific integrity of the project. The investigators will use an existing DSMB in the Department of Psychiatry at Case Western Reserve University (CWRU) chaired by a psychiatrist who is independent from the investigators. Other members of the DSMB include a doctoral level statistician, a data analyst, and an experienced member of the department research staff. All members will be independent from the sponsor and competing interests.

The Chair of the DSMB will conduct real-time monitoring of all serious adverse events (SAE) that occur. SAEs are defined as events that result in any of the following: death, a life threatening experience, inpatient hospitalization or prolongation of existing hospitalization, a persistent or significant disability/incapacity, or a congenital anomaly/birth defect (or an event that may require medical or surgical intervention to prevent one of the outcomes listed above). The relationship of the SAE to study participation will be determined by the PIs. This assessment will occur within 24 h of notification of the SAE. For those situations where the SAE is determined to be study related, the DSMB Chair will review the SAE within 24 h and determine whether it is expected or unexpected. The DSMB Chair will ensure that the SAE is reported to the IRB according to established IRB guidelines. The DSMB meets at least semi-annually to review all adverse events.

The PIs will hold weekly meetings with study staff to review study progress and any issues that may come up regarding adverse events. A summary report of all adverse events will be submitted to the National Heart, Lung, and Blood Institute annually and at the end of the study. Adverse events will be identified by the study investigators and/or qualified research assistants. All adverse events, whether considered serious or not, will be recorded and reviewed by the study PIs on an ongoing basis, and reported to the IRB according to local IRB policy.

Given that this is deemed a minimal risk trial, there will be no interim analyses or stopping guidelines. There will be no post-trial care or compensation for those who suffer harm from trial participation. All participants will continue with their providers during and following the trial. Any participant at risk for imminent harm to self or others will be handled on a case by case basis. Participants will be terminated from the trial if deemed unsafe to continue in study procedures by the PI or the participant’s clinician (such as for worsening condition or increased harm risk), if the participant requests to be removed or withdraws consent, or if the participant is lost to follow-up. Since the intervention is an addition to their regular treatment and does not include medication, there will be no follow-up with research team.

### Data dissemination

Following completion of the last trial participant, results regarding the primary outcomes of the trial will be submitted for publication within 1 year of the final study visit. All final peer-reviewed manuscripts that arise from this proposal will be submitted to the digital archive PubMed Central. The data generated in this grant will be presented at national or international conferences and published in a timely fashion in journals in the field of primary care, HTN, psychiatry, behavioral medicine, BD, adherence, and behavioral trials, among others. This will ensure that the appropriate audience has access to the findings of the trial. Similarly, data from the trial will be presented at conferences focused on primary care, behavioral medicine, and psychiatry.

## Discussion

This project is innovative in that it focuses on common chronic health conditions in a high-risk group of individuals, those with poorly controlled HTN and comorbid mental illness. This innovation uses a technology-facilitated novel health promotion approach with promising pilot data and a theoretical model which views sustained medication-taking behavior in the context of habit formation. The study represents a first-ever adherence-based RCT specifically targeting BP and mental health symptom control in people with BD and includes implementation elements in the study design in order to inform future scale-up.

Individuals with BD tend to have erratic lifestyles, and the present intervention is not dependent on frequent contact between provider and patient. Rates of medical clinic attendance for people with BD are often poor and iTAB-CV addresses this problem by administering the intervention on a mobile phone [79]. Persons of lower socioeconomic status often use a mobile phone over a landline phone because of frequent address changes and adults living in poverty represent a greater proportion of cellphone-only households [80]. In the pilot data with the target population, 73.7% were African Americans, 73.7% were on Medicaid, and 87% of the sample had a cellphone. The majority of effort in this texting intervention is in the initial programming of the automated system. Once the script is in place, the intervention can be extended for longer periods of time, modified to increase the pool of queries and reminders, and altered to include additional medications.

Because the intervention asks the participant to respond to reminders and queries, the intervention is interactive unlike an alarm clock or other programmed devices. Previous studies show that the interactive properties of texting were often so well accepted that some participants elected to receive texts after the study ended [50]. iTAB-CV is flexible and can send reminders consistent with the participant’s schedule. If medications are switched during the study, iTAB-CV can be updated. The iTAB-CV intervention is highly scalable within and across patient populations.

In summary, this proposal addresses an important but understudied area: how to improve BP control in people with BD. A novel intervention that uses a model of habit formation, remediates for prospective cognitive or planning deficits, is customized, addresses both psychotropic and non-psychotropic non-adherence, and is delivered via mobile phone has the potential to improve physical and emotional health. Promising preliminary data on iTAB-CV suggests that it is feasible, highly acceptable and effective. This trial has the potential to advance care for this high-risk population and will be amenable to broad scale-up for other challenging comorbid populations.

## Trial status

We used the protocol version approved on April 21, 2021, and anticipate starting recruitment for the clinical trial in June of 2021, and completing recruitment by the end of 2024.

## Supplementary Information

**Additional file 1.**

**Additional file 2.**

## Data Availability

The authors of this paper at CWRU will have full access to the final trial dataset. Authors at UCSD will have access to de-identified data. The de-identified dataset analyzed for the current study and statistical code are available from the corresponding author on reasonable request, as is the full protocol.

## Abbreviations

iTAB-CV: Individualized Texting for Adherence Building – Cardiovascular
RCT: Randomized controlled trial
CV: Cardiovascular
BD: Bipolar disorder
SBP: Systolic blood pressure
HTN: Hypertension
SPRINT: Systolic Blood Pressure Intervention Trial
AHA: American Heart Association
SM: Self-monitoring
BP: Blood pressure
IRB: Institutional Review Board
SAB: Stakeholders Advisory Board
REDCap: Research Electronic Data Capture
BHS: British Hypertension Society
MI: Mobile interventionist
RA: Research assistant
TRQ: Tablets Routine Questionnaire
BPRS: Brief Psychiatric Rating Scale
MADRS: Montgomery-Asberg Depression Rating Scale
MASES-R: Medication Adherence Self-Efficacy Scale
SRHI: Self-Report Habit Index
BMQ: Beliefs about Medicines Questionnaire
AMSQ: Attitudes toward Mood Stabilizers Questionnaire
MINI: Mini-International Neuropsychiatric Interview
CCI: Charlson Comorbidity Index
REALM-R: Rapid Estimate of Adult Literacy in Medicine Revised
RMASS: Repeated Measures and Sample Size
AR(1): Autoregressive
CWRU: Case Western Reserve University
MAR: Missing at random
DSMB: Data Safety and Monitoring Board
SAE: Serious adverse events

## References

[1] Weiner M, Warren L, Fiedorowicz JG (2011). Cardiovascular morbidity and mortality in bipolar disorder. Ann Clin Psychiatry.

[2] Fleischhacker WW, Cetkovich-Bakmas M, De Hert M, Hennekens CH, Lambert M, Leucht S (2008). Comorbid somatic illnesses in patients with severe mental disorders: clinical, policy, and research challenges. J Clin Psychiatry.

[3] Kessler RC, Berglund P, Demler O, Jin R, Merikangas KR, Walters EE (2005). Lifetime prevalence and age-of-onset distributions of DSM-IV disorders in the National Comorbidity Survey Replication. Arch Gen Psychiatry.

[4] Kessler RC, Chiu WT, Demler O, Merikangas KR, Walters EE (2005). Prevalence, severity, and comorbidity of 12-month DSM-IV disorders in the National Comorbidity Survey Replication. Arch Gen Psychiatry.

[5] Sajatovic M (2012). Complexities of care in bipolar disorder. Psychiatr Ann.

[6] Merikangas KR, Akiskal HS, Angst J, Greenberg PE, Hirschfeld RM, Petukhova M (2007). Lifetime and 12-month prevalence of bipolar spectrum disorder in the National Comorbidity Survey replication. Arch Gen Psychiatry.

[7] Cloutier M, Greene M, Guerin A, Touya M, Wu E (2018). The economic burden of bipolar I disorder in the United States in 2015. J Affect Disord.

[8] Birkenaes AB, Opjordsmoen S, Brunborg C, Engh JA, Jonsdottir H, Ringen PA (2007). The level of cardiovascular risk factors in bipolar disorder equals that of schizophrenia: a comparative study. J Clin Psych.

[9] Ramsey CM, Leoutsakos JM, Mayer LS, Eaton WW, Lee HB (2010). History of manic and hypomanic episodes and risk of incident cardiovascular disease: 11.5 year follow-up from the Baltimore epidemiologic catchment area study. J Affect Disord.

[10] Ramsey CM, Spira AP, Mojtabai R, Eaton WW, Roth K, Lee HB (2013). Lifetime manic spectrum episodes and all-cause mortality: 26-year follow-up of the NIMH epidemiologic catchment area study. J Affect Disord.

[11] Westman J, Hallgren J, Wahlbeck K, Erlinge D, Alfredsson L, Osby U. Cardiovascular mortality in bipolar disorder: a population-based cohort study in Sweden. BMJ Open. 2013;3(4). 10.1136/bmjopen-2012-002373 PMC3641504 23604348

[12] Goldstein BI, Schaffer A, Wang S, Blanco C (2015). Excessive and premature new-onset cardiovascular disease among adults with bipolar disorder in the US NESARC cohort. J Clin Psychiatry.

[13] Goldstein BI, Fagiolini A, Houck P, Kupfer DJ (2009). Cardiovascular disease and hypertension among adults with bipolar I disorder in the United States. Bipolar Disord.

[14] Costa FV (1996). Compliance with antihypertensive treatment. Clin Exp Hypertens.

[15] Cramer JA, Benedict A, Muszbek N, Keskinaslan A, Khan ZM (2008). The significance of compliance and persistence in the treatment of diabetes, hypertension and dyslipidaemia: a review. Int J Clin Pract.

[16] Corrao G, Parodi A, Nicotra F, Zambon A, Merlino L, Cesana G (2011). Better compliance to antihypertensive medications reduces cardiovascular risk. J Hypertens.

[17] Perreault S, Dragomir A, Roy L, White M, Blais L, Lalonde L (2010). Adherence level of antihypertensive agents in coronary artery disease. Br J Clin Pharmacol.

[18] Kettani FZ, Dragomir A, Cote R, Roy L, Berard A, Blais L (2009). Impact of a better adherence to antihypertensive agents on cerebrovascular disease for primary prevention. Stroke.

[19] Dragomir A, Cote R, Roy L, Blais L, Lalonde L, Berard A (2010). Impact of adherence to antihypertensive agents on clinical outcomes and hospitalization costs. Med Care.

[20] Sajatovic M, Biswas K, Kilbourne AK, Fenn H, Williford W, Bauer MS (2008). Factors associated with prospective long-term treatment adherence among individuals with bipolar disorder. Psychiatr Serv.

[21] Levin JB, Krivenko A, Howland M, Schlachet R, Sajatovic M (2016). Medication adherence in patients with bipolar disorder: a comprehensive review. CNS Drugs.

[22] Chang CW, Sajatovic M, Tatsuoka C (2015). Correlates of attitudes towards mood stabilizers in individuals with bipolar disorder. Bipolar Disord.

[23] Devulapalli KK, Ignacio RV, Weiden P, Cassidy KA, Williams TD, Safavi R (2010). Why do persons with bipolar disorder stop their medication?. Psychopharmacol Bull.

[24] Whelton PK, Carey RM, Aronow WS, Casey DE, Collins KJ, Dennison Himmelfarb C (2018). 2017 ACC/AHA/AAPA/ABC/ACPM/AGS/APhA/ASH/ASPC/NMA/PCNA guideline for the prevention, detection, evaluation, and management of high blood pressure in adults: a report of the American College of Cardiology/American Heart Association task force on clinical practice guidelines. J Am Coll Cardiol.

[25] Wright JT, Williamson JD, Whelton PK, Snyder JK, Sink KM, Rocco MV (2015). A randomized trial of intensive versus standard blood-pressure control. N Engl J Med.

[26] Baldessarini RJ, Perry R, Pike J (2008). Factors associated with treatment nonadherence among US bipolar disorder patients. Hum Psychopharmacol.

[27] Velligan DI, Weiden PJ, Sajatovic M, Scott J, Carpenter D, Ross R (2009). The expert consensus guideline series: adherence problems in patients with serious and persistent mental illness. J Clin Psychiatry.

[28] Levin JB, Krivenko A, Bukach A, Tatsuoka C, Cassidy KA, Sajatovic M (2017). A reexamination of nonpsychiatric medication adherence in individuals with bipolar disorder and medical comorbidities. J Nerv Ment Dis.

[29] Daugherty SL, Powers JD, Magid DJ, Masoudi FA, Margolis KL, O’Connor PJ (2012). The association between medication adherence and treatment intensification with blood pressure control in resistant hypertension. Hypertension..

[30] Algabbani FM, Algabbani AM (2020). Treatment adherence among patients with hypertension: findings from a cross-sectional study. Clin Hypertens.

[31] Viswanathan M, Golin CE, Jones CD, Ashok M, Blalock S, Wines RC (2012). Closing the quality gap: revisiting the state of the science (vol. 4: medication adherence interventions: comparative effectiveness). Evid Rep/Technol Assessment.

[32] Nieuwlaat R, Wilczynski N, Navarro T, Hobson N, Jeffery R, Keepanasseril A (2014). Interventions for enhancing medication adherence. Cochrane Database Syst Rev.

[33] Lee E, Xiang YT, Man D, Au RW, Shum D, Tang WK (2010). Prospective memory deficits in patients with bipolar disorder: a preliminary study. Arch Clin Neuropsychol.

[34] Zogg JB, Woods SP, Sauceda JA, Wiebe JS, Simoni JM (2012). The role of prospective memory in medication adherence: a review of an emerging literature. J Behav Med.

[35] Bogner HR, de Vries HF, O’Donnell AJ, Morales KH (2013). Measuring concurrent oral hypoglycemic and antidepressant adherence and clinical outcomes. Am J Manag Care.

[36] Sajatovic M, Levin J, Fuentes-Casiano E, Cassidy KA, Tatsuoka C, Jenkins JH (2011). Illness experience and reasons for nonadherence among individuals with bipolar disorder who are poorly adherent with medication. Compr Psychiatry.

[37] Mohr DC, Spring B, Freedland KE, Beckner V, Arean P, Hollon SD (2009). The selection and design of control conditions for randomized controlled trials of psychological interventions. Psychother Psychosom.

[38] Tucker KL, Sheppard JP, Stevens R, Bosworth HB, Bove A, Bray EP (2017). Self-monitoring of blood pressure in hypertension: a systematic review and individual patient data meta-analysis. PLoS Med.

[39] Chan AW, Tetzlaff JM, Gøtzsche PC, Altman DG, Mann H, Berlin JA (2013). SPIRIT 2013 explanation and elaboration: guidance for protocols of clinical trials. BMJ..

[40] British Hypertension Society. Home blood pressure monitoring protocol. 2017. https://bihsoc.org/wp-content/uploads/2017/09/Protocol.pdf. Accessed 19 Dec 2020.

[41] Omron Healthcare. Clinical validation. 2021. https://omronhealthcare.com/service-and-support/clinical-validation/. Accessed 19 Dec 2020.

[42] Levin JB, Sams J, Tatsuoka C, Cassidy KA, Sajatovic M. Use of automated medication adherence monitoring in bipolar disorder research: pitfalls, pragmatics, and possibilities. Therapeutic advances. Psychopharmacology. 2015;2045125314566807. 10.1177/2045125314566807 PMC4521443 26240747

[43] NIH NIMH. Bipolar disorder. 2015. [https://www.nimh.nih.gov/health/publications/bipolar-disorder/index.shtml. Accessed 19 Dec 2020.

[44] Bolman C, Arwert TG, Vollink T (2011). Adherence to prophylactic asthma medication: habit strength and cognitions. Heart Lung.

[45] Blixen C, Sajatovic M, Moore DJ, Depp C, Cushman C, Cage J (2018). Patient participation in the development of a customized m-health intervention to improve medication adherence in poorly adherent individuals with bipolar disorder (BD) and hypertension (HTN). Int J Healthcare.

[46] Amrhein PC, Miller WR, Yahne CE, Palmer M, Fulcher L (2003). Client commitment language during motivational interviewing predicts drug use outcomes. J Consult Clin Psychol.

[47] Cole-Lewis H, Kershaw T (2010). Text messaging as a tool for behavior change in disease prevention and management. Epidemiol Rev.

[48] Moore D, Poquette A, Casaletto K, Gouaux B, Montoya J, Posada C (2015). Individualized texting for adherence building (iTAB): improving antiretroviral dose timing among HIV-infected persons with co-occurring bipolar disorder. AIDS Behavior.

[49] Rami B, Popow C, Horn W, Waldhoer T, Schober E (2006). Telemedical support to improve glycemic control in adolescents with type 1 diabetes mellitus. Eur J Pediatr.

[50] Richmond SJ, Keding A, Hover M, Gabe R, Cross B, Torgerson D (2015). Feasibility, acceptability and validity of SMS text messaging for measuring change in depression during a randomised controlled trial. BMC Psychiatry.

[51] Shimbo D, Artinian NT, Basile JN, Krakoff LR, Margolis KL, Rakotz MK (2020). Self-measured blood pressure monitoring at home: a joint policy statement from the American Heart Association and American Medical Association. Circulation..

[52] Scott J, Pope M (2002). Nonadherence with mood stabilizers: prevalence and predictors. J Clin Psychiatry.

[53] Adams J, Scott J (2000). Predicting medication adherence in severe mental disorders. Acta Psychiatr Scand.

[54] Levin JB, Sams J, Tatsuoka C, Cassidy KA, Sajatovic M (2015). Use of automated medication adherence monitoring in bipolar disorder research: pitfalls, pragmatics, and possibilities. Ther Adv Psychopharmacol.

[55] Sheehan DV, Lecrubier Y, Sheehan KH, Amorim P, Janavs J, Weiller E (1998). The Mini-international neuropsychiatric interview (M.I.N.I.): the development and validation of a structured diagnostic psychiatric interview for DSM-IV and ICD-10. J Clin Psychiatry.

[56] Chaudhry S, Jin L, Meltzer D (2005). Use of a self-report-generated Charlson comorbidity index for predicting mortality. Med Care.

[57] Arozullah AM, Yarnold PR, Bennett CL, Soltysik RC, Wolf MS, Ferreira RM (2007). Development and validation of a short-form, rapid estimate of adult literacy in medicine. Med Care.

[58] Scott J, Pope M (2002). Self-reported adherence to treatment with mood stabilizers, plasma levels, and psychiatric hospitalization. Am J Psychiatry.

[59] Overall JA, Gorham DR (1962). The brief psychiatric rating scale. Psychol Rep.

[60] Leentjens AF, Verhey FR, Lousberg R, Spitsbergen H, Wilmink FW (2000). The validity of the Hamilton and Montgomery-Asberg depression rating scales as screening and diagnostic tools for depression in Parkinson’s disease. Int J Geriatr Psychiatry.

[61] Fernandez S, Chaplin W, Schoenthaler AM, Ogedegbe G (2008). Revision and validation of the medication adherence self-efficacy scale (MASES) in hypertensive African Americans. J Behav Med.

[62] Verplanken B, Orbell S (2003). Reflections on past behavior: a self-report index of habit strength. J Appl Soc Psychol.

[63] Horne R, Weinman J, Hankins M (1999). The beliefs about medicines questionnaire: the development and evaluation of a new method for assessing the cognitive representation of medication. Psychol Health.

[64] Harvey NS (1991). The development and descriptive use of the lithium attitudes questionnaire. J Affect Disord.

[65] Harris PA, Taylor R, Thielke R, Payne J, Gonzalez N, Conde JG (2009). Research electronic data capture (REDCap)-a metadata-driven methodology and workflow process for providing translational research informatics support. J Biomed Inform.

[66] Stata Corp (2013). Stata statistical software: release 13.

[67] Bland JM, Altman DG (1986). Statistical methods for assessing agreement between two methods of clinical measurement. Lancet..

[68] MacKinnon D (2008). Introduction to statistical mediation analysis.

[69] Preacher KJ, Hayes AF (2008). Asymptotic and resampling strategies for assessing and comparing indirect effects in multiple mediator models. Behav Res Methods.

[70] Imai K, Keele L, Yamamoto T (2010). Identification, inference and sensitivity analysis for causal mediation effects. Stat Sci.

[71] Wang W, Nelson S, Albert JM (2013). Estimation of causal mediation effects for a dichotomous outcome in multiple-mediator models using the mediation formula. Stat Med.

[72] RMASS. Statistical power computations for 3-level mixed-effects regression models [computer program]. University of Illinois at Chicago.

[73] Sajatovic M, Gunzler DD, Kanuch SW, Cassidy KA, Tatsuoka C, McCormick R (2017). A 60-week prospective RCT of a self-management intervention for individuals with serious mental illness and diabetes mellitus. Psychiatr Serv.

[74] Ogedegbe G, Chaplin W, Schoenthaler A, Statman D, Berger D, Richardson T (2008). A practice-based trial of motivational interviewing and adherence in hypertensive African Americans. Am J Hypertens.

[75] Wong GW, Wright JM. Blood pressure lowering efficacy of nonselective beta-blockers for primary hypertension. Cochrane Database Syst Rev. 2014;(2):CD007452. 10.1002/14651858.CD007452.pub2 PMC10603273 24585007

[76] Rubin DB (1976). Inference and missing data. Biometrika..

[77] Ten Have TR, Kunselman AR, Pulkstenis EP, Landis JR (1998). Mixed effects logistic regression models for longitudinal binary response data with informative drop-out. Biometrics..

[78] Chen F, editor. Missing no more: using the MCMC procedure to model missing data. SAS Global Forum; 2013.

[79] Krishna S, Boren SA, Balas EA (2009). Healthcare via cell phones: a systematic review. Telemed J E Health.

[80] Blumberg S, Luke J. Wireless substitution: early release of estimates from the National Health Interview Survey, July-December 2013. InNCH Stats. 2014.

