# Supplementary material for: Using mHealth to improve adherence and reduce blood pressure in individuals with hypertension and bipolar disorder (iTAB-CV): study protocol for a 2-stage randomized clinical trial
Source: Trials. 2022 Jun 29;23:539. doi: 10.1186/s13063-022-06449-9 (PMC9244195; doi:10.1186/s13063-022-06449-9)
Supplement: Supplementary file 1 — Additional file 1. [file 13063_2022_6449_MOESM1_ESM.pdf]

IRB NUMBER: STUDY20200330
IRB APPROVAL DATE: 8/9/2021
IRB EFFECTIVE DATE: 8/13/2021
IRB EXPIRATION DATE: 3/8/2022

UNIVERSITY HOSPITALS CLEVELAND MEDICAL CENTER
CONSENT FOR INVESTIGATIONAL STUDIES
(v. 10.2019)

**Project Title:** Using mHealth to improve adherence and reduce blood pressure in individuals with hypertension and bipolar disorder (iTAB-CV RCT)	
**Principal Investigator:** Jennifer Levin, Ph.D.	

Key Information: The following is a short summary of this study to help you decide whether or not to be a part of this study. More detailed information is listed later on in this form.

Why am I being invited to take part in a research study?
You are being asked to participate in this study because you have hypertension (high blood pressure) and bipolar disorder and have recently missed taking some medication.

Things I should know about a research study

- Someone will explain this research study to you.
- Whether or not you take part is up to you.
- You can choose not to take part.
- You can agree to take part and later change your mind.
- Your decision will not be held against you.
- You can ask all the questions you want before you decide.

Introduction/Purpose
You are being asked to participate in a research study about how to help people with high blood pressure and bipolar disorder remember to take their medication. You are being asked to participate in this study because you have high blood pressure and bipolar disorder and have recently missed taking some medication. Doctors at University Hospitals Cleveland Medical Center (UH) and Case Western Reserve University (CWRU) Department of Psychiatry want to find out if sending text messages, an intervention called Individualized Texting for Adherence Building - Cardiovascular (iTAB-CV) might help patients remember to take their medication.

This study will take place at UH. It is funded by the National Heart, Lung and Blood Institute (NHLBI) and Dr. Levin is the study principal investigator. A total of 200 people will participate in this study. Half of the people who participate in this study will receive the iTAB-CV intervention in addition to Self- Monitoring and the other half will receive just Self-Monitoring. All participants will continue under the care of their prescribing clinicians.

Before you decide whether or not to participate in this study, you must be told the purpose of the research study, how this study may help you, any risks to you, and what is expected of you. This process is called informed consent. You will be asked to sign this consent form before you can participate in the study. Please make sure you fully understand what this study is about and ask any questions you might have. If you decide to participate in this study a copy of this consent form will be given to you for your records.

The purpose of this study is to find out if certain text messages will help people to take their medications as they are prescribed.

Key Study Procedures

Your participation in this study will last approximately 12 months. During that time, you will be asked to come in for five in person study/research visits. You will also complete 3 study/research visits via phone/video platform (month 3, month 5 and month 9). At each visit, you will be asked if you have missed any doses of your medications. At each in person visit, you will be asked about your mental health symptoms and your attitudes and how you are taking your medication. You will also have your blood pressure taken. More detailed information about the study procedures can be found under "Detailed Study Procedures".

Key Risks

Some of the questions you are asked may be upsetting, or you may feel uncomfortable answering them. If you do not wish to answer a question, you may skip it and go to the next question. You may feel tired after completing all the questionnaires.

Although your condition may improve during the study, it is also possible that your condition may worsen during the study.

The study requires you to place a device on top of a standard pill bottle (eCAP) which may affect the use of pill organizers or other devices to manage your medications. It is possible that this may affect your medication taking behavior.

Even though every effort will be made to protect your confidentiality, there is a risk of loss of confidentiality.

More detailed information about the risks of this study can be found under "Detailed Risks"

Benefits

This study may or may not benefit you. Information obtained in this study may help improve care for other patients with bipolar disorder and high blood pressure.

Alternatives to Study Participation

While participating in this study you will continue to receive your usual treatment, including prescriptions from your regular mental healthcare provider. Therefore, the alternative to participating in this study is to not participate.

Detailed Information: The following is more detailed information about this study in addition to the information listed above.

Detailed Study Procedures

Your participation in this study will last approximately 12 months. During that time, you will be asked to come in for five in person study/research visits (screening, baseline/month 2, month 4, month 6 and month 12). You will also complete 3 study/research visits via phone/video platform (3 months, 5 months and 9 months).

If you are not able to come in for an in-person visit, you may be able to complete study procedures over the internet (video conferencing) or by phone. These remote visits may be audio or video recorded for quality assurance purposes.

At the screening visit, you will sign the informed consent form and you will undergo a psychiatric diagnostic evaluation. You will be asked basic demographic questions, such as your age and gender. Your height, weight, and blood pressure will also be taken. If you are completing this visit remotely, you will be asked to take your own blood pressure 2 times consecutively, twice a day (in the AM and in the PM) for 3 to 7 days. Once we have obtained 12 readings total (6 in the AM and 6 in the PM), you will no longer need to do this. The study will provide you with a blood pressure monitor so you can do this throughout the study. In addition, you will be asked questions about your attitudes, mental health symptoms, and how you are taking your medication. You will also receive an eCAP for the medication you take for high blood pressure which will record time and date of bottle opening. The screening visit will last about 90-120 minutes.

Two months later, you will be asked to come in for a baseline/month 2 visit. At this visit, you will be asked questions about your attitudes, mental health symptoms, and how you are taking your medication. Your blood pressure will be measured again. If you are completing this visit remotely, you will be asked to take your own blood pressure 2 times consecutively, twice a day (in the AM and in the PM) for 3 to 7 days. Once we have obtained 12 readings total (6 in the AM and 6 in the PM), you will no longer need to do this. You will also watch a 13-minute educational video about the symptoms, risks, and the important role of medication in the treatment of both high blood pressure and bipolar disorder.

Once you complete the first part of the baseline visit, you will be randomly assigned by chance (like the flip of a coin) to either: 1) receive the iTAB-CV intervention in addition to using the eCAP, and taking your blood pressure every week, or 2) just use the eCAP, take your blood pressure every week, and rate your mood once a week via a text message. You will have a 50% change of receiving the iTAB-CV intervention. If you are in the group that tests the intervention,

a structured interview with the study staff will also be conducted. This will last 30-45 minutes and will occur separately from the first part of your baseline visit The structured interview will determine the following: preferred name, schedule of high blood pressure and bipolar disorder medications, and description of high blood pressure and bipolar disorder medications. This information will be used to create personalized text reminders. You will begin receiving texts about the role of medicine in treating high blood pressure and bipolar disorder starting a day after the baseline visit and will receive texts once a day. You will also be asked to rate your mood once a week through a text message. You will be taught how to answer the text messages. If you are in the group that does not test the iTAB-CV intervention, study staff will just teach you how to answer the text messages. The discussion you have with study staff to set up text messages for both groups will be audio/video recorded. The baseline visit will last about 120 minutes.

At month 4, month 6 and month 12, you will return to the office and will be asked questions about your attitudes, mental health symptoms, and how you are taking your medication. Your blood pressure will be measured again. If you are completing this visit remotely, you will be asked to take your own blood pressure 2 times consecutively, twice a day (in the AM and in the PM) for 3 to 7 days. Once we have obtained 12 readings total (6 in the AM and 6 in the PM), you will no longer need to do this. If you are completing visits remotely, you will report your blood pressure reading and weight to us. At month 4 and month 6, you will also answer questions about your experience with the study intervention. These visits will last approximately 45-60 minutes.

If you are in the iTAB-CV group, at your month 4 visit, you will also be randomly assigned by chance (like the flip of a coin) to either start off with 1 text per day and gradually decrease to 1 text per week or to receive one text a week for 8 weeks. You have a 50% chance of being in the group that gradually tapers to once a week and a 50% chance of being in the group that receives texts once a week for that period.

At month 3, month 5 and month 9, you will speak to a member of the study staff over the phone or via video platform to discuss how you are taking your medication, measure blood pressure, and scan your eCAP. .

Detailed Risks

Some of the questions you are asked may be upsetting. You may feel uncomfortable answering some questions. If you do not wish to answer a question, you may skip it and go to the next question. You may feel tired after completing all the questionnaires.

Your condition may improve during the study but it is also possible that your condition may worsen during the study. Your regular mental health care provider and your regular primary care providers

will continue to monitor your conditions while you are in the study. Whether or not your condition improves or worsens while you are in the study, your regular care providers and you will continue to make decisions about your care. These decisions may or may not include no longer participating the study.

Should your condition unexpectedly worsen and you report plans to harm yourself, for your own safety, you may be evaluated by the principal investigator or another mental health professional for possible hospitalization.

The study requires you to place a device on top of a standard pill bottle (eCAP) which may affect the use of pill organizers or other devices to manage your medications. It is possible that this may affect your medication taking behavior.

Even though every effort will be made to protect your confidentiality, there is a risk of loss of confidentiality. It is possible that you could put personal information on the device that could link you to the delivered text messages, but the risk of losing confidential information in this way is the same as the risk of owning and using any cellular phone. Reminders do not use medication names and mood questions are general. You will get to decide what words you would like to use to refer to bipolar disorder and high blood pressure. The text messages will be delivered from a phone number that will not be linked to the specific study goals.

Financial Information

Depending on completion of screening visit, you will receive up to $30 for the screening visit and $30 for the next three in person study visits you complete. You will receive $40 for completing the month 12 study visit. You will also receive $10 for each phone visit you complete. If you complete your visits in person, you will receive an additional $10 for bringing your eCAP to the month 2, month 4, month 8 and month 12 visits. If you complete your month 2, month 4, month 8 and month 12 visits remotely, you will receive this $10 for uploading your data to the app on your phone and transmitting it to the site during your remote visit. If your phone is not compatible with the app and you cannot upload your data to the app, you will be given up to $40, upon return of the eCAP, to account for visits in which you were not able to scan your eCAP. If you end the study before your month 12 visit and return your eCAP, you will receive $10. If you end study prior to randomization, and return your blood pressure monitor, you will receive $10. If you complete all study visits and bring your eCAP to all in-person visits/upload data to the app during remote visits, you will receive $230 total.

To receive payment you must agree to complete a W-9 form, which requires you to provide an address and social security number to the accounting department. This payment to you may be

considered taxable income by the IRS. You will be issued a 1099-Misc form only if payment exceeds $600 from all studies in which you are participating, in a fiscal year.
If necessary, transportation to and from your study visits will be provided in the form of a bus ticket or parking voucher. We will not be covering the costs associated with your cellular phone, including text messaging.

There is no cost to you or your insurance for participation in this research study.

Contact for Future Research
Our study team may have additional research studies in the future. We would like your permission to contact you in the future if we think you could be a potential participant in one of our studies. Please check one of the boxes below that indicates your choice to be contacted for future research.

- ☐ Please contact me by _________ for future research opportunities.

- ☐ Please do not contact me for future research opportunities.

Research-Related Injury
In the event you suffer a research related injury as a result of being in this study, University Hospitals is available to provide medical treatment for such injury. Provision of such medical treatment does not imply any negligence or other wrongdoing on the part of University Hospitals, the Sponsor or any of the physicians or other study personnel. If you believe that you have been injured as a result of participating in the study, please immediately contact the Principal Investigator or your study doctor at University Hospitals. If you cannot reach the Principal Investigator or your study doctor, do not delay treatment. You may seek treatment by another doctor. If you are seen or treated by a doctor other than the Principal Investigator or your study doctor, you should inform such doctor that you are in this study and, if possible, take this document with you to assist with your treatment. Always contact the Principal Investigator or your study doctor to alert them of any treatment you receive for an injury or illness you experience during this Study.

The costs for medical treatment as a result of a research related injury may be billed to you or your medical insurance plan, if applicable. Medical insurance plans may or may not cover costs for medical treatment of research related injuries. If you have insurance, you should check with your medical insurance plan before deciding to participate in this research study. In the event your medical insurance plan covers some or all of the treatment costs, you may still be responsible for co-pays or deductibles as required by your medical insurance plan.

Neither Sponsor nor University Hospitals has set aside any money to pay you or to pay for your treatment if you suffer a research related injury as a result of being in the study. There are no plans for University Hospitals or Sponsor to provide other forms of compensation (such as lost wages or other indirect losses) to you for research related injuries. You are not waiving any legal rights by signing this form, including the right to seek compensation for an injury. To help avoid injury, it is very important to follow all study directions.

Clinical Trial Information

U.S. NATIONAL INSTITUTES OF HEALTH (NIH) CLINICAL TRIAL DATABASE: A description of this clinical trial will be available on http:///www.clinicaltrials.gov, as required by U.S. Law. This website will not include information that can identify you. At most, the website will include a summary of the results. You can search this website at any time to find out information about the trial and basic results.

Student/Employee Rights

Choosing not to participate or withdrawing from this study will not affect your employment or class standing, nor will the results be shared with your supervisor.

Termination of Participation

You may decide at any time to stop participating in this study. There are no consequences to you for withdrawing from the study. Your participation in this study may be discontinued by the study doctor if she feels it would be in your best interest.

Confidentiality

Any information obtained during this study and identified with you will remain confidential and will be disclosed only with your permission. All information will be coded to protect your confidentiality and then stored in cabinets in the locked offices of the investigators or on the UHCMC secure server. Information that is collected is subject to review by the National Heart, Lung, and Blood Institute (NHLBI), the Institutional Review Board, and the Office of Human Research Protection. These entities may also have access to your files.

If identifiers are removed from your identifiable private information or identifiable samples that are collected during this research, that information or those samples could be used for future research studies or distributed to another investigator for future research studies without your additional informed consent.

All videotapes, audiotapes, and photographs will be destroyed at the end of the study. You will be asked to sign a separate consent form called GM-23 that allows us to use this information. If you

do not agree to being recorded and sign the GM-23, the investigators will need to determine whether you will be able to participate in the study or not.

Certificate of Confidentiality
This research is covered by a Certificate of Confidentiality from the National Institutes of Health. The researchers with this Certificate may not disclose or use information, documents, or biospecimens that may identify you in any federal, state, or local civil, criminal, administrative, legislative, or other action, suit, or proceeding, or be used as evidence, for example, if there is a court subpoena, unless you have consented for this use. Information, documents, or biospecimens protected by this Certificate cannot be disclosed to anyone else who is not connected with the research except, if there is a federal, state, or local law that requires disclosure (such as to report child abuse or communicable diseases but not for federal, state, or local civil, criminal, administrative, legislative, or other proceedings, see below); if you have consented to the disclosure, including for your medical treatment; or if it is used for other scientific research, as allowed by federal regulations protecting research subjects.

The Certificate cannot be used to refuse a request for information from personnel of the United States federal or state government agency sponsoring the project that is needed for auditing or program evaluation by National Heart, Lung, and Blood Institute (NHLBI), which is funding this project or for information that must be disclosed in order to meet the requirements of the federal Food and Drug Administration (FDA). You should understand that a Certificate of Confidentiality does not prevent you from voluntarily releasing information about yourself or your involvement in this research. If you want your research information released to an insurer, medical care provider, or any other person not connected with the research, you must provide consent to allow the researchers to release it.

The Certificate of Confidentiality will not be used to prevent disclosure as required by federal, state, or local law of intent to harm yourself or others, or of actual or suspected child abuse or neglect.

The Certificate of Confidentiality will not be used to prevent disclosure for any purpose you have consented to in this informed consent document such as to another provider.

Privacy of Protected Health Information (HIPAA)
The Health Insurance Portability & Accountability Act (HIPAA) is a Federal law that helps to protect the privacy of your health information and to whom this information may be shared within and outside of University Hospitals. This Authorization form is specifically for a research study entitled "Using mHealth to improve adherence and reduce blood pressure in individuals

with hypertension and bipolar disorder (iTAB-CV RCT)" and will tell you what health information (called Protected Health Information or PHI) will be collected for this research study, who will see your PHI and in what ways they can use the information. In order for the Principal Investigator, Jennifer Levin, Ph.D., and the research study staff to collect and use your PHI, you must sign this authorization form. You will receive a copy of this signed Authorization for your records. If you do not sign this form, you may not join this study. Your decision to allow the use and disclosure of your PHI is voluntary and will have no impact on your treatment at University Hospitals. By signing this form, you are allowing the researchers for this study to use and disclose your PHI in the manner described below.

Generally the Principal Investigator and study staff at University Hospitals and Case Western Reserve University who are working on this research project will know that you are in a research study and will see and use your PHI. The researchers working on this study will collect the following PHI about you:

- your name, initials, address, telephone number, email address, date of birth and other demographic information;
- your medical history (including the history and diagnosis of your disease and your family medical history) and the name of your physician(s) and locations where you received any treatment;
- information about other medical conditions that may affect your participation, including information relating to mental health, behavioral health and psychiatric disorders; and alcohol and drug dependence or abuse;
- specific information about any treatment/therapy you receive while participating in the research study and treatment you received prior to the research study (including treatments and therapies, surgeries, hospitalizations and medications);
- information on side effects from the medicine you take or have taken and how these side effects were treated;
- information about how frequent you take your medications;
- information about your general health status and the status of your disease or medical condition; and
- numbers or codes that identify you such as your social security number, medical record number, and research study case number.

This PHI will be used to determine if an educational and behavioral intervention program is helpful in the treatment of patients with bipolar disorders and hypertension. Your access to your PHI may be limited during the study to protect the study results.

Your PHI may also be shared with the following groups/persons associated with this research study or involved in the review of research: Case Western Reserve University, including the Department of Psychiatry, Department of Neurology, Department of Medicine; University of California San Diego (UCSD); other staff from the Principal Investigator's medical practice group; University Hospitals, including the Center for Clinical Research and the Law Department; Government representatives or Federal agencies, when required by law. It is possible, that in the future, additional research sites may be added. In this event, your PHI that was collected during this research project may be shared with research personnel at these additional sites.

Your permission to use and disclose your PHI does not expire. However, you have the right to change your mind at any time and revoke your authorization. If you revoke your authorization, the researchers will continue to use the information that they previously collected, but they will not collect any additional information. Also, if you revoke your authorization you may no longer be able to participate in the research study. To revoke your permission, you must do so in writing by sending a letter to: Jennifer Levin, Ph.D., Department of Psychiatry, University Hospitals Cleveland Medical Center, 10524 Euclid Ave., Cleveland, OH 44106. If you have a complaint or concerns about the privacy of your health information, you may also write to the UH Privacy Officer, Management Service Center, 3605 Warrensville Center, MSC 9105, Shaker Heights, OH 44122 or to the Federal Department of Health and Human Services (DHHS) at DHHS Regional Manager, Office of Civil Rights, US Department of Health and Human Services Government Center, JF Kennedy Federal Building, Room 1875, Boston, MA 02203. Complaints should be sent within 180 days of finding out about the problem.

The researchers and staff agree to protect your health information by using and disclosing it only as permitted by you in this Authorization and as directed by state and Federal law. University Hospitals is committed to protecting your confidentiality. Please understand that once your PHI has been disclosed to anyone outside of University Hospitals, there is a risk that your PHI may no longer be protected; however other Federal and State laws may provide continued protection of your information.

Summary of Your Rights as a Participant in a Research Study

Your participation in this research study is voluntary. Refusing to participate will not alter your usual health care or involve any penalty or loss of benefits to which you are otherwise entitled. If you decide to join the study, you may withdraw at any time and for any reason without penalty or loss of benefits. If information generated from this study is published or presented, your identity will not be revealed. In the event new information becomes available that may affect the risks or benefits associated with this study or your willingness to participate in it, you will be notified so that you can decide whether or not to continue participating. If you experience

physical injury or illness as a result of participating in this research study, medical care is available at University Hospitals Cleveland Medical Center (UHCMC) or elsewhere; however, UHCMC has no plans to provide free care or compensation for lost wages.

Disclosure of Your Study Records

Efforts will be made to keep the personal information in your research record private and confidential, but absolute confidentiality cannot be guaranteed. The University Hospitals Cleveland Medical Center Institutional Review Board may review your study records. If this study is regulated by the Food and Drug Administration (FDA), there is a possibility that the FDA might inspect your records. In addition, for treatment studies, the study sponsor and possibly foreign regulatory agencies may also review your records. If your records are reviewed your identity could become known.

Contact Information

______________________________ has described to you what is going to be done, the risks, hazards, and benefits involved. The Principal Investigator, Jennifer Levin, Ph.D., can also be contacted at 216-844-5057. If you have any questions, concerns or complaints about the study in the future, you may also contact them later.

If the researchers cannot be reached, or if you would like to talk to someone other than the researcher(s) about; concerns regarding the study; research participant's rights; research- related injury; or other human subject issues, please call the University Hospitals Cleveland Medical Center's Research Subject Rights phone line at (216) 983-4979 or write to: The Associate Chief Scientific Officer, The Center for Clinical Research, University Hospitals Cleveland Medical Center, 11100 Euclid Avenue, Lakeside 1400, Cleveland, Ohio, 44106-7061.

Signature

Signing below indicates that you have been informed about the research study in which you voluntarily agree to participate; that you have asked any questions about the study that you may have; and that the information given to you has permitted you to make a fully informed and free decision about your participation in the study. By signing this consent form, you do not waive any legal rights, and the investigator(s) or sponsor(s) are not relieved of any liability they may have. A copy of this consent form will be provided to you.

x	

Signature of Participant		Date	Time
x			
Printed Name of Participant			

x			
Signature of person obtaining informed consent		Date	Time
x			
Printed name of person obtaining informed consent			
