# Supplementary material for: Using mHealth to improve adherence and reduce blood pressure in individuals with hypertension and bipolar disorder (iTAB-CV): study protocol for a 2-stage randomized clinical trial
Source: Trials. 2022 Jun 29;23:539. doi: 10.1186/s13063-022-06449-9 (PMC9244195; doi:10.1186/s13063-022-06449-9)
Supplement: Supplementary file 2 — Additional file 2. [file 13063_2022_6449_MOESM2_ESM.pdf]

UNIVERSITY HOSPITALS CLEVELAND MEDICAL CENTER
CONSENT FOR INVESTIGATIONAL STUDIES
(v. 7.2020)

**Project Title:** Using mHealth to improve adherence and reduce blood pressure in individuals with hypertension and bipolar disorder (iTAB-CV RCT)<br>Stakeholder Advisory Board (SAB) Members	
**Principal Investigator:** Jennifer Levin, Ph.D.	

Key Information: The following is a short summary of this study to help you decide whether or not to be a part of this study. More detailed information is listed later on in this form.

Why am I being invited to take part in a research study?
You are being asked to participate in this stakeholder advisory board because you have high blood pressure and bipolar disorder, you are a family member of someone who has high blood pressure and bipolar disorder, you provide clinical care to patients who have high blood pressure and/or bipolar disorder, or you are health system administration representative who interacts with patients who have high blood pressure and/or bipolar disorder.

Things I should know about a research study
- Someone will explain this research study to you.
- Whether or not you take part is up to you.
- You can choose not to take part.
- You can agree to take part and later change your mind.
- Your decision will not be held against you.
- You can ask all the questions you want before you decide.

Introduction/Purpose
The purpose of this research is to help people with high blood pressure and bipolar disorder remember to take their medication. Doctors at University Hospitals Cleveland Medical Center (UH) and Case Western Reserve University (CWRU) Department of Psychiatry want to find out if sending text messages, an intervention called Individualized Texting for Adherence Building - Cardiovascular (iTAB-CV) might help patients remember to take their medication. You will be one of up to 12 participants enrolled in this study at CWRU/UH.

Key Study Procedures
We expect that you will be in this research study for up to 5 years. During that time, you will be asked to attend three stakeholder advisory board (SAB) meetings in the first 6 months of the project and then once yearly for a total of 7 meetings over the course of 5 years. Each meeting will last about 60 minutes. During these meetings, we will get your input on the iTAB-CV intervention, help us determine how best to enroll participants in to the randomized controlled trial (RCT) portion of the study and help us determine how the iTAB-CV intervention might be used in clinical settings in the future. You will attend these meetings remotely via the internet and/or phone. More detailed information about the study procedures can be found under "Detailed Study Procedures".

Key Risks
It is possible that some of the questions you are asked may be upsetting, or you may feel uncomfortable answering them. If you do not wish to answer a question, you may skip it. More detailed information about the risks of this study can be found under "Detailed Risks"

Benefits
We cannot promise any benefits to you or others from your taking part in this research. However, you may find it helpful to participate in the group and discuss your experiences with others.

Alternatives to Study Participation
Participation in research is completely voluntary. You can decide to participate or not to participate. Your alternative to participating in this research study is to not participate.

Detailed Information: The following is more detailed information about this study in addition to the information listed above.

Detailed Study Procedures
If you agree to participate in this research, we would ask you to do the following things:

1. Sign the informed consent form (10-20 minutes)
2. Complete a demographics form (up to 5 minutes). This form will be completed either on paper or in REDCap via a survey link sent your email address.
3. Participate in three advisory board meetings in the first 6 months of the project and then once yearly for a total of 7 meetings over the course of 5 years. (about 60-90 minutes each meeting)
4. Complete a satisfaction survey after each meeting (up to 5 minutes).

You will be video and audio recorded while you are in the advisory board meetings.

You can choose to stop participating for any reason at any time. However, if you decide to stop participating in the study, we encourage you to tell the researchers.

All SAB meetings will take place remotely via the internet and/or phone.

Detailed Risks
Some of the activities we will ask you to complete might make you feel uncomfortable or tired and some of the questions we ask may be upsetting, or you may feel uncomfortable answering

them. If you do not wish to complete an activity or answer a question, you may skip it and go to the next question, take a break, or stop your participation in this study at any time.
If you are a person who receives treatment for bipolar disorder and high blood pressure, since you will meet other individuals who have bipolar disorder and high blood pressure, individuals who are family members of someone with bipolar disorder and high blood pressure, or work with people who have bipolar disorder and/or hypertension, there may be some loss of confidentiality and other people will find out that you receive treatment for bipolar disorder and hypertension. All participants will be reminded that it is very important to respect the privacy and confidentiality of everybody in the group.

Financial Information

There is no cost to you or your insurance for participation in this study.

You will receive $30 for each advisory board meeting you attend. If you attend all 7 meetings, you will receive $210 over the five years. Payment will be made by check mailed to you after the meeting is completed. If you do not have a bank account to cash the check, the research assistant will discuss alternate payment methods with you.

To receive payment you must agree to complete a W-9 form which requires you to provide an address and social security number to the accounting department. This payment to you may be considered taxable income by the IRS. You will be issued a 1099-Misc form only if payment exceeds $600 from all studies in which you are participating, in a fiscal year.

Student/Employee Rights

Choosing not to participate or withdrawing from this study will not affect your employment or class standing, nor will the results be shared with your supervisor.

Termination of Participation

You may decide at any time to stop participating in this study. There are no consequences to you for withdrawing from the study.

Confidentiality

The records of this research will be kept confidential. Any time information is collected, there is a potential risk for loss of confidentiality. Every effort will be made to keep your information confidential; however, this cannot be guaranteed.

In any sort of report we might publish, we will not include any information that will make it possible to identify a participant. Research records will be kept in a locked file and access will

be limited to the researchers, the institutional review board responsible for protecting human participants, and regulatory agencies.
If identifiers are removed from your identifiable private information or identifiable samples that are collected during this research, that information or those samples could be used for future research studies or distributed to another investigator for future research studies without your additional informed consent.

Data without any identifiable information may be shared with our collaborators on this study at the University of California San Diego.

All videotapes, audiotapes, and photographs will be destroyed at the end of the study. You will be asked to sign a separate consent form called GM-23 that allows us to use this information. If you do not agree to being recorded and do not sign the GM-23 you will not be allowed to participate in this study.

Privacy of Protected Health Information (HIPAA)
The Health Insurance Portability & Accountability Act (HIPAA) is a Federal law that helps to protect the privacy of your health information and to whom this information may be shared within and outside of University Hospitals. This Authorization form is specifically for a research study entitled "Using mHealth to improve adherence and reduce blood pressure in individuals with hypertension and bipolar disorder (iTAB-CV RCT)" and will tell you what health information (called Protected Health Information or PHI) will be collected for this research study, who will see your PHI and in what ways they can use the information. In order for the Principal Investigator, Jennifer Levin, Ph.D., and the research study staff to collect and use your PHI, you must sign this authorization form. You will receive a copy of this signed Authorization for your records. If you do not sign this form, you may not join this study. Your decision to allow the use and disclosure of your PHI is voluntary and will have no impact on your treatment at University Hospitals. By signing this form, you are allowing the researchers for this study to use and disclose your PHI in the manner described below.

Generally the Principal Investigator and study staff at University Hospitals and Case Western Reserve University who are working on this research project will know that you are in a research study and will see and use your PHI. The researchers working on this study will collect the following PHI about you:

- Your name, initials, address, telephone number, date of birth and other demographic information;
- Numbers or codes that identify you such as your social security number, medical record number, and research study case number.

This PHI will be used to refine the iTAB-CV intervention and how iTAB-CV may be used in a clinical setting. Your access to your PHI may be limited during the study to protect the study results.

Your PHI may also be shared with the following groups/persons associated with this research study or involved in the review of research: Case Western Reserve University, including the Department of Psychiatry, Department of Neurology, Department of Medicine, other staff from the Principal Investigator's medical practice group; University Hospitals, including the Center for Clinical Research and the Law Department; any UH or CWRU employee required to process information for research, finance, compliance, or hospital operation, and Government representatives or Federal agencies, when required by law. It is possible, that in the future, additional research sites may be added. In this event, your PHI that was collected during this research project may be shared with research personnel at these additional sites.

Your permission to use and disclose your PHI does not expire. However, you have the right to change your mind at any time and revoke your authorization. If you revoke your authorization, the researchers will continue to use the information that they previously collected, but they will not collect any additional information. Also, if you revoke your authorization you may no longer be able to participate in the research study. To revoke your permission, you must do so in writing by sending a letter to Jennifer Levin, Ph.D., Department of Psychiatry, University Hospitals Cleveland Medical Center, 10524 Euclid Ave., Cleveland, OH 44106; If you have a complaint or concerns about the privacy of your health information, you may also write to the UH Privacy Officer, Management Service Center, 3605 Warrensville Center, MSC 9105, Shaker Heights, OH 44122 or to the Federal Department of Health and Human Services (DHHS) at DHHS Regional Manager, Office of Civil Rights, US Department of Health and Human Services Government Center, JF Kennedy Federal Building, Room 1875, Boston, MA 02203. Complaints should be sent within 180 days of finding out about the problem.

The researchers and staff agree to protect your health information by using and disclosing it only as permitted by you in this Authorization and as directed by state and Federal law. University Hospitals is committed to protecting your confidentiality. Please understand that once your PHI has been disclosed to anyone outside of University Hospitals, there is a risk that your PHI may no longer be protected; however other Federal and State laws may provide continued protection of your information.

Summary of Your Rights as a Participant in a Research Study

Your participation in this research study is voluntary. Refusing to participate will not alter your usual health care or involve any penalty or loss of benefits to which you are otherwise entitled.

If you decide to join the study, you may withdraw at any time and for any reason without penalty or loss of benefits. If information generated from this study is published or presented, your identity will not be revealed. In the event new information becomes available that may affect the risks or benefits associated with this study or your willingness to participate in it, you will be notified so that you can decide whether or not to continue participating. If you experience physical injury or illness as a result of participating in this research study, medical care is available at University Hospitals Cleveland Medical Center (UHCMC) or elsewhere; however, UHCMC has no plans to provide free care or compensation for lost wages.

Disclosure of Your Study Records

Efforts will be made to keep the personal information in your research record private and confidential, but absolute confidentiality cannot be guaranteed. The University Hospitals Cleveland Medical Center Institutional Review Board may review your study records. If this study is regulated by the Food and Drug Administration (FDA), there is a possibility that the FDA might inspect your records. In addition, for treatment studies, the study sponsor and possibly foreign regulatory agencies may also review your records. If your records are reviewed your identity could become known.

Contact Information

_______________________________ has described to you what is going to be done, the risks, hazards, and benefits involved. The Principal Investigator Jennifer Levin, Ph.D. can also be contacted 216-844-5057. If you have any questions, concerns or complaints about the study in the future, you may also contact them later.

If the researchers cannot be reached, or if you would like to talk to someone other than the researcher(s) about; concerns regarding the study; research participant's rights; research- related injury; or other human subject issues, please call the University Hospitals Cleveland Medical Center's Research Subject Rights phone line at (216) 983-4979 or write to: The Associate Chief Scientific Officer, The Center for Clinical Research, University Hospitals Cleveland Medical Center, 11100 Euclid Avenue, Lakeside 1400, Cleveland, Ohio, 44106-7061.

Signature

Signing below indicates that you have been informed about the research study in which you voluntarily agree to participate; that you have asked any questions about the study that you may have; and that the information given to you has permitted you to make a fully informed and free decision about your participation in the study. By signing this consent form, you do not waive

any legal rights, and the investigator(s) or sponsor(s) are not relieved of any liability they may have. A copy of this consent form will be provided to you.

X		
Signature of Participant	Date	Time
X		
Printed Name of Participant		

X		
Signature of person obtaining informed consent	Date	Time
X		
Printed name of person obtaining informed consent		
